# A theory of consciousness from a theoretical computer science perspective: Insights from the Conscious Turing Machine

**DOI:** 10.1073/pnas.2115934119

**Published:** 2022-05-20

**Authors:** Lenore Blum, Manuel Blum

**Affiliations:** ^a^Computer Science Department, Carnegie Mellon University, Pittsburgh, PA 15213;; ^b^Electrical Engineering and Computer Science (EECS), University of California, Berkeley, CA 94720

**Keywords:** theoretical computer science, consciousness, artificial intelligence, global workspace, complexity theory

## Abstract

This paper examines consciousness from the perspective of theoretical computer science (TCS), a branch of mathematics concerned with understanding the underlying principles of computation and complexity, including the implications and surprising consequences of resource limitations. We propose a formal TCS model, the Conscious Turing Machine (CTM). The CTM is influenced by Alan Turing's simple yet powerful model of computation, the Turing machine (TM), and by the global workspace theory (GWT) of consciousness originated by cognitive neuroscientist Bernard Baars and further developed by him, Stanislas Dehaene, Jean-Pierre Changeux, George Mashour, and others. Phenomena generally associated with consciousness, such as blindsight, inattentional blindness, change blindness, dream creation, and free will, are considered. Explanations derived from the model draw confirmation from consistencies at a high level, well above the level of neurons, with the cognitive neuroscience literature.

## Significance

This paper provides evidence that a theoretical computer science (TCS) perspective can add to our understanding of consciousness by providing a simple framework for employing tools from computational complexity theory and machine learning. Just as the Turing machine is a simple model to define and explore computation, the Conscious Turing Machine (CTM) is a simple model to define and explore consciousness (and related concepts). The CTM is not a model of the brain or cognition, nor is it intended to be, but a simple substrate-independent computational model of (the admittedly complex concept of) consciousness. This paper is intended to introduce this approach, show its possibilities, and stimulate research in consciousness from a TCS perspective.

The quest to understand consciousness, once the purview of philosophers and theologians, is now actively pursued by scientists of many stripes.* We study consciousness from the perspective of theoretical computer science (TCS), a branch of mathematics concerned with understanding the underlying principles of computation and complexity. This abstract theory has provided both a theoretical foundation for the computer revolution and (also) surprising new concepts and ingenious applications stemming from considerations of computational complexity. It deals with the consequences and unexpected insights that come from taking resource limitations into account. We claim that the perspective and unique insights of TCS add to the understanding of consciousness and related concepts, such as free will.

## 1. A TCS Perspective on Consciousness

Our view is that consciousness is a property of all properly organized computing systems, whether made of flesh and blood or metal and silicon. With this in mind, we give a simple abstract substrate-independent computational model of consciousness (section 2). We are not looking to model the brain nor to suggest neural correlates of consciousness, interesting as they are. We are looking to understand consciousness and its related phenomena.

We begin this introduction with a brief overview of TCS, its perspective, and an example of a relevant seemingly paradoxical concept that got defined and understood by TCS. We then outline how the perspective of TCS informs our model and understanding of consciousness. Following the Introduction, we present the Conscious Turing Machine (CTM), which is our formalization of a variant of the global workspace theory (GWT). The reader who wants the formal model first can go directly to section 2.

### 1.1. TCS.

Alan Turing’s seminal paper “On computable numbers, with an application to the Entscheidungsproblem” (32) is arguably the genesis of TCS. That paper presents a mathematical definition of a “computing machine,” now known as the Turing machine (TM). In it, Turing defines a simple theoretical universal programmable computer that can compute any function computable by any computer or supercomputer.^†^

Theorems are the raison d’etre of mathematical theories, and Turing proves what might be called the first theorem of TCS, namely the unsolvability of the Halting problem. In modern parlance, this theorem proves there can be no universal (debugging) program for determining which computer programs halt and which do not; it is just not possible to construct one.

The unsolvability of the Halting problem is equivalent to the undecidability of elementary number theory (35), and implies a weak form of Kurt Gödel’s first incompleteness theorem (36).^‡^

After Gödel and Turing, mathematical logicians started categorizing which problems were solvable and which were not as well as investigating the esoteric hierarchy of unsolvable problems.

With the advent and wider availability of computing machines in the 1960s, it soon became clear that a number of important problems that were solvable in principle could not in fact be solved, not even with the fastest conceivable computers, and that this was not a problem with the state of technology but something deeper.^§^

Researchers in the emerging field of TCS [notably Jack Edmonds (38), Stephen Cook (39), Richard Karp (40), and Leonid Levin (41)] realized that among natural finite (and therefore, solvable) problems, there appeared to be a dichotomy between those problems that were feasibly (efficiently) solvable and those that were not, mirroring the earlier dichotomy between solvable and unsolvable. Feasibly solvable became formalized mathematically as solvable (by some computer program) in polynomial time (P). Furthermore, the realization emerged that problems solvable in polynomial time and problems checkable in polynomial time (NP) might not be equivalent.^¶^ Indeed, deciding the equivalence would answer the famous million dollar P = ?NP question (42).

Besides defining a hierarchy of serial fast (poly time) computational complexity classes, TCS defines a hierarchy of parallel superfast (polylog time) computational complexity classes. Both hierarchies inform the definitions and choices employed in our model. Michael Sipser’s book (43) is a great introduction to TCS.

Understanding the dichotomy between easy and hard, quick and slow, and their implications launched a complexity revolution with a rich theory, reframing of ideas, novel concepts, and stunning applications. Indeed, developments in computational complexity over the past 40 y have shown how to use hardness to our advantage to deal with seemingly impossible problems.

We illustrate with the (relevant) concept of a computer-generated random sequence, called a pseudorandom sequence.

On the face of it, the very idea of a pseudorandom sequence is so incongruous that von Neumann (44) joked that “[a]nyone who considers arithmetical methods of producing random digits is, of course, in a state of sin.”

More precisely, a pseudorandom sequence generator is a feasible (polynomial time) computer program for generating sequences that cannot be distinguished from truly random sequences (generated by independent tosses of a fair coin) by any feasible computer program. Thus, in the polynomial time world in which we live, pseudorandom sequences are, for all intents and purposes, truly random. This understanding was impossible without the clarifications made by TCS, including the distinctions between polynomial and superpolynomial complexity (45).

An application of the above ideas is to replace the use of random sequences in the (probabilistic) CTM by sequences produced by pseudorandom generators supplied with (short) random seeds. In particular, if the probabilistic CTM has “free will,” as will be argued, then so does this deterministic variant of CTM. The free will of this deterministic CTM is counter to some (perhaps much) of the thinking on determinism (e.g., ref. 46).

### 1.2. Now for Consciousness.

The TCS perspective is employed in defining the CTM, a simple machine that formalizes mathematically (and modifies with dynamics) the GWT of consciousness originated by cognitive neuroscientist Bernard Baars (14) and extended by Dehaene (15) and Mashour et al. (47) in their global neuronal workspace theory (GNWT). In *In the Theater of Consciousness*, Baars (16) describes consciousness through a theater analogy as the activity of actors in a play performing on the stage of working memory, their performance under observation by a huge audience of unconscious processors sitting in the dark.

In the CTM, the stage of GWT is represented by short-term memory (STM) that at any moment in time contains CTM’s conscious content. The audience members are represented by enormously powerful processors—each with its own expertise—that make up CTM’s long-term memory (LTM) (section 2.1.1). These LTM processors make predictions and get feedback from CTM’s world. Based on this feedback, learning algorithms internal to each processor improve that processor’s behavior (section 2.6).

LTM processors, each with their own specialty, compete (sections 2.1.2 and 2.3) to get their questions, answers, and information in the form of chunks (section 2.1.3) on the stage for immediate broadcast to the audience.

Conscious awareness—elsewhere called attention—is defined formally in the CTM as the reception by the LTM processors of the broadcast of CTM’s conscious content. In time, some of these processors become connected via links (section 2.1.4) that turn conscious communication (through STM) into unconscious communication (through links) between these LTM processors. Communication via links about a broadcasted chunk reinforces its conscious awareness, a process that Dehaene, and Changeux (10) call ignition.

While these definitions are natural, they are merely definitions; they do not provide a proof that the CTM feels conscious. We argue, however, that these definitions, together with explanations derived from the CTM model, capture commonly accepted intuitive concepts of consciousness and agree, at a high level, with cognitive neuroscience explanations of phenomena generally associated with consciousness.

Although inspired by Baars’ GWT architecture, the CTM integrates additional features essential for its feeling of consciousness. These include its dynamics, its rich multimodal inner language (which we call Brainish) (section 2.2), and special LTM processors that enable it to create models of the world (sections 3 and 4).

### 1.3. Complexity Considerations.

The consequences of limited resources play a crucial role in our high-level explanations for consciousness-related phenomena, such as change blindness and the feeling of free will.

They also enter into fixing the detailed definition of CTM. These details include, for example,

1) the formal definition of a chunk, which is the information that each LTM processor puts into the competition for consciousness at every tick of the clock (section 2.1.3);
2) the fast probabilistic competition algorithm that selects which one of the many competing chunks reaches consciousness (sections 2.3 and 2.4); and
3) the machine learning algorithm (section 2.6) in each processor that uses feedback from global broadcasts, other processors, and the outside world to update its processor’s competitiveness and reliability.

Although inspired by Turing’s simple yet powerful model of a computer, the CTM is not a standard TM. That is because what gives the CTM its “feeling of consciousness” is not its computing power nor its input–output maps but rather, its global workspace architecture, its predictive dynamics (cycles of prediction, feedback, and learning), its rich multimodal inner language, and certain special LTM processors such as the Model of the World processor.

As we have said, we are not looking for a model of the brain but for a simple model of consciousness, and even there, the CTM model can hardly be expected to explain everything; it is too simple for that. The reasonableness of the model (and its TCS perspective) should be judged by its contribution to the discussion and understanding of consciousness and related “hard” problems.

This paper presents an overview of the CTM model; we refer the reader to refs. 48 and 49 for additional details. Whereas ref. 48 explores explanations for the feelings of pain and pleasure in the CTM, this paper presents additional phenomena generally associated with consciousness. We start (in section 4) with three examples related to vision (blindsight, inattentional blindness, and change blindness) and then, follow with a discussion of dreams and free will. We give explanations derived from the model and draw confirmation from consistencies at a high level with the cognitive neuroscience literature.^#^ Confirmation for the model also comes from agreement with aspects of other theories of consciousness.

In what follows, statements about the CTM are in roman. Statements particular to humans or animals are generally in italics.

## 2. CTM Model Overview

### 2.1. Basic CTM Structure and Definitions of Consciousness in the CTM.

We assume that the CTM has a finite lifetime **T**. Time is measured in discrete clock ticks, t = 0, 1, 2, …, **T**∼ 10^10^ (roughly 10 ticks per second, *that being the alpha rhythm of the brain*). The CTM is born at time 0.

The CTM is a seven-tuple,

**< STM, LTM, Up Tree, Down Tree, Links, Input, Output >**

with the following components.

#### 2.1.1. STM and LTM processors.

In the CTM, **STM** is a small memory capable of holding a single **chunk**, defined in section 2.2. LTM is a (massive) collection of **N** processors, **N** > 10^7^, with each processor being a random access machine with a random access memory large enough to hold a small multiple of **T** chunks. Processors are in **LTM** only, not in STM, so when we say processor, we mean **LTM** processor. Certain special LTM processors are particularly responsible for CTM’s feeling of consciousness. These include especially a Model of the World processor, an Inner Speech processor, and other Inner generalized Speech processors for handling inner vision, inner tactile sensation, and so on (section 3).

#### 2.1.2. The Up Tree competition and Down Tree broadcast.

The **Down Tree** is a simple down-directed tree of height 1 with a single root in STM and **N** edges directed from that root to the leaves, one leaf in each LTM processor.

The **Up Tree** is an up-directed binary tree of height h with **N** leaves, one leaf in each LTM processor, and a (single) root in STM. LTM processors, each with their own specialty, compete via the **Up Tree competition** (section 2.3) to get their questions, answers, and information into STM for immediate **broadcast** via the Down Tree to the audience of all LTM processors. For simplicity in CTM, all LTM processors submit information to the competition for STM, and all processors receive all broadcasts from STM. *In humans, however, the dorsal stream of vision is never conscious (never gets to STM); only the ventral stream is conscious (53).*

*This bottom-up/top-down cycle is analogous to the global neuronal workspace (GNW) hypothesis (17) that “conscious access proceeds in two successive phases … In a first phase, lasting from ≈100 to ≈300 ms, the stimulus climbs up the cortical hierarchy of processors in a primarily bottom-up and nonconscious manner. In a second phase, if the stimulus is selected for its adequacy to current goals and attention state, it is amplified in a top-down manner and becomes maintained by sustained activity of a fraction of GNW neurons, the rest being inhibited. The entire workspace is globally interconnected in such a way that only one such conscious representation can be active at any given time.”*

#### 2.1.3. Chunks, conscious content, conscious awareness, and stream of consciousness.

Questions, answers, and information are conveyed in the form of **chunks** (defined formally in section 2.2). The chunk that wins the Up Tree competition to get into STM is called the **conscious content** of CTM.

In the CTM, unlike Baars’ theater, there is always exactly one and the same actor in STM (the stage).^ǁ^ At every step in time, that actor gets handed the winning chunk as a script for immediate broadcast via the Down Tree. We say that CTM becomes **consciously aware** of this content when it is received by all LTM processors via this broadcast.**

We have defined conscious awareness as the reception by all LTM processors of STM’s broadcast rather than the appearance in STM of the winning chunk to emphasize that the feeling of consciousness arises after processors, including especially the Model of the World and Inner Speech, receive the broadcast.

Our definition of conscious awareness in the CTM aligns roughly with what cognitive neuroscientists call “attention” (for example, refs. 18 and 47). What we call the feeling of consciousness in the CTM (section 3) aligns roughly with what cognitive neuroscientists call “awareness” or “subjective consciousness.”

CTM is constantly bubbling with the activity of chunks competing for STM, its winners being (constantly) broadcast from STM to LTM. The time-ordered chunks that are broadcast from STM to LTM form a **stream of consciousness**. This stream, as argued in section 3, is part of the subjective feeling of consciousness.

#### 2.1.4. Links, unconscious communication, and global ignition.

All communications between processors initially occur via STM. For example, processor A can submit a query to the Up Tree competition for STM. If the query wins the competition, it is broadcast to all LTM processors. Processor B may then submit an answer via the competition, which if it wins, gets broadcast and so on.

If A acknowledges that B’s answer is useful sufficiently often, then a bidirectional **link** forms between A and B. *This linking is reminiscent of the Hebbian principle (56) that “[n]eurons that fire together wire together.”*

In addition to processors sending chunks to the Up Tree competition, processors send chunks over links. In this way, **conscious communication** (through STM) between A and B can turn into direct **unconscious communication** by chunks being sent (through links) between A and B.^††^ As additional links form between A and B, we say that the link between A and B is strengthened.

Links are channels for transmitting information between processors. Those chunks sent between linked processors following the broadcast of CTM’s conscious content can reenforce and sustain conscious awareness. *This reenforcement is related to what Dehaene and Changeux (10) call “global ignition” in their GNWT. As Dehaene (15) writes, “Global ignition … occurs when a broadcast excitation exceeds a threshold and becomes self-reinforcing, with some neurons exciting others that, in turn, return the excitation. The connected [cells] burst into a self-sustained state of high level activity, a reverberating ‘cell assembly,’ as Hebb called it.”*

#### 2.1.5. Input and Output maps: Sensors and actuators.

CTM’s **environment** (Env) is a subset of R^m^(t), where R denotes the real numbers, m is a positive integer dimension, and t (a nonnegative integer) is time. **Input maps** take (time-varying) environmental information acquired by CTM’s **sensors** (which for simplicity, we assume are part of the input maps) and send it to designated LTM processors that convert the environmental information into chunks (section 2.2). **Output maps** take command information from LTM processors to **actuators** (which we assume are part of the output maps) to act on the environment.

#### 2.1.6. Summary of connections.

**Fig. 1.**
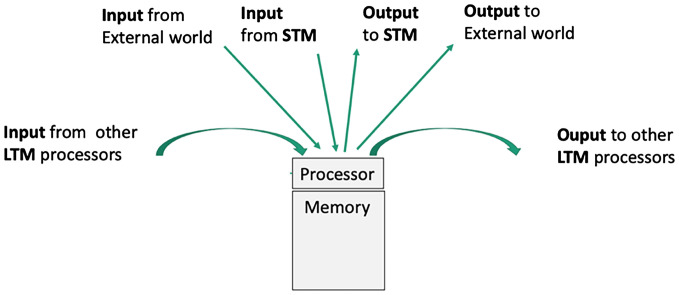
Connections in the CTM to and from an LTM processor.

In summary, there are five kinds of connections in the CTM that provide paths and mechanisms for transmitting information. The five, also shown in Fig. 1 (connections in the CTM to and from an LTM processor), are

1) Env → LTM: directed edges from the environment via sensors to processors of the sensory data;
2) LTM → STM: via the Up Tree;
3) STM → LTM: via the Down Tree;
4) LTM → LTM: bidirectional edges (links) between processors; and
5) LTM → Env: directed edges from specific processors (like those that generate instructions for finger movement) to the environment via actuators (like the fingers that receive instructions from these processors) that act on the environment (via the actions of the fingers from these processors).

### 2.2. Brainish (the CTM’s Multimodel Inner Language), Gists, and Chunks.

**Brainish** is CTM’s inner language used to communicate between processors whether via the competition and broadcasts or directly through links. On the other hand, languages used internally by processors vary in general from one processor to another; they include but are not restricted to Brainish.

Brainish is the language used to express inner speech, inner vision, inner sensations, imaginings, and dreams. It includes coded representations of inputs and outputs all expressed with succinct multimodal Brainish words and phrases called gists. A gist can hold the essence of a scene or the (high-level expandable) idea of a proof. It can be an answer to a query, an insight of some sort, a dream image, a (description of) pain, and so on. Brainish is able to express and manipulate images, sounds, tactile sensations, and thoughts—including unsymbolized thoughts—better than outer languages, like English, Chinese, or “Doggish” (see ref. 57). We claim that having an expressive inner language is an important component of the feeling of consciousness (section 3).

Information is carried on all edges, between processors, between STM and LTM, from input to LTM, and from LTM to output by chunks.

A **chunk** is a six-tuple

**< address, t, gist, weight, intensity, mood >**,

where

**address** is the address of the LTM processor that produced the chunk, **t** is the time that the chunk was produced, **gist** is the information “concisely expressed” in Brainish that the processor intends to communicate, **weight** is a valenced number that the processor gives the gist, and **intensity** and **mood** start off (at time t) as **|weight|** and **weight**, respectively.

We note that the size of the chunk (and hence, the size of its components, including its gist) will necessarily be bounded by computational complexity considerations (section 2.4 and ref. 48 have more specifics).

### 2.3. The (Probabilistic) Up Tree Competition: The Coin-Flip Neuron and Competition Function.

The **Up Tree competition** is the mechanism that determines which LTM processor will get its chunk into STM. At each clock tick t = 0, 1, …, **T**, the t^th^ competition starts with each processor p putting its chunk into the node that is the processor’s leaf of the Up Tree. After a chunk is submitted to the Up Tree competition and while it moves up the "competition tree", its address, t, gist, and weight remain unchanged, but its intensity and mood get updated to incorporate ever more global information.

Deciding whether or not a chunk moves up a level or drops out is made by a fast tiny parallel circuit, with one such circuit located in each of the nonleaf nodes of the Up Tree, each making its decision in one clock tick (the time between two successive clock ticks).

In the **probabilistic Up Tree competition**, which we discuss here, each node of the Up Tree has and uses a **coin-flip neuron** in its built-in circuit. A coin-flip neuron is a device that takes as input a (ordered) pair (a, b) of nonnegative real numbers (a ≥ 0 and b ≥ 0) and in one step, does the following:

if a > 0 or b > 0, it outputs a with probability a/(a + b), else b; if a = b = 0, it outputs a with probability 1/2, else b.

At each clock tick, the circuit in a nonleaf node v runs a local competition that probabilistically selects one of v’s two (sibling) children based on a comparison of the chunks they contain and then moves (a variant of) the chosen chunk into v. That chunk is said to be the **winner of the local competition** at/for v.

The local competition employs the Up Tree's **competition function** f, a function that maps chunks to nonnegative real numbers in a fraction of a clock tick, to choose the **local winner**.

Specifically, suppose at level s, 0 < s ≤ h, and node v_s_ at that level, chunk_p(L)_ and chunk_p(R)_ are the chunks in v_s_’s left and right children, respectively. Then,

1) with probability {f(chunk_p(L)_)/(f(chunk_p(L)_) + f(chunk_p(R)_)) if the denominator ≠ 0 or with probability 1/2 if the denominator = 0}, chunk_p(L)_ is the local winner;
2) else chunk_p(R)_ is the local winner.

We now specify the chunk that moves into v_s_. Suppose that the local winner at v_s_ is the variant of chunk_p,t,0_ having address_p_, t, gist_p,t,0_, and weight_p,t,0_. Then, the chunk that moves into v_s_ will be

$$c\mathrm{hun}k_{p,t,s}=<a\mathrm{ddres}s_{p},t,\;gist_{p,t,s},w\mathrm{eigh}t_{p,t,s},i\mathrm{ntensit}y_{p,t,s},mood_{p,t,s}>,\text{where}$$

gist_p,t,s_ = gist_p,t,0_; weight_p,t,s_ = weight_p,t,0_; intensity_p,t,s_ = (intensity_p(L),t,s−1_) + (intensity_p(R),t,s−1_); and mood_p,t,s_ = (mood_p(L),t,s−1_) + (mood_p(R),t,s−1_).

Note that intensity_p,t,s_ = ∑_p’_ (intensity_p’,t,0_) and mood_p,t,s_ = ∑_p’_ (mood_p’,t,0_), where the two sums ∑_p’_ are over all LTM processors p’ in the subtree rooted at v_s_.

*Remark 1*: Updating the chunk at node v_s_ consists of computing the probabilities needed to select the local winner and then, to make the needed modifications of its intensity and mood. This must all be done in one clock tick. This puts bounds on both the amount of computation that can be performed in a node and on the size of the chunk in that node (section 2.4 and ref. 48 have more specifics).

By a simple induction, the winner of the Up Tree competition (the conscious content of CTM at time t + h) will look like

$$c\mathrm{hun}k_{p,t,h}=<a\mathrm{ddres}s_{p},t,gist_{p,t,0},w\mathrm{eigh}t_{p,t,0},i\mathrm{ntensit}y_{p,t,h},mood_{p,t,h}>,$$

where intensity_p,t,h_ = ∑_all **N** processors p’ in LTM_ (intensity_p’,t,0_) and mood_p,t,s_ = ∑_all **N** processors p’ in LTM_ (mood_p’,t,0_).

Let t ≥ h. The current mood of CTM at time t, mood_t_, is defined to be the mood of the chunk that is broadcast from STM at time t. Thus, CTM becomes consciously aware of mood_t_ at time t + 1.

*Remark 2*: mood_t_ = ∑_all **N** LTM processors p_ mood_p,t-h,0_, so mood_t_/**N** is the average mood of the chunks submitted to the competition at time t **−** h.

**mood_t_** is a measure of CTM’s “optimism/happiness” if positive, or “pessimism/sadness” if negative at time t. *[In humans, “emotion is always valenced—either pleasant or unpleasant—and dependent on the pleasure system” (58).]*

**intensity_t_** is a measure of CTM’s level of “energy/enthusiasm/confidence” at time t.

We say that a competition function f is **additive** if f(chunk_p,t,s_(v_s_)) = f(chunk_L_(v_s_)) + f(chunk_R_(v_s_)). Examples of additive competition functions include f(chunk_p,t,s)_ = intensity_p,t,s_ or more generally,

$$f(c\mathrm{hun}k_{p,t,s})=i\mathrm{ntensit}y_{p,t,s}+\;c\cdot mood_{p,t,s}\;\text{for}\;\text{any}\;\text{real}\;c,-1\leq c\leq +1,\text{but}\;\text{not}\;f(c\mathrm{hun}k_{p,t,s})=|mood_{p,t,s}|.$$

We call a CTM with a probabilistic Up Tree competition a **probabilistic CTM**. In this paper, unless otherwise stated, all CTMs will from now on be probabilistic.

Theorem. If the competition function f of a probabilistic CTM is additive, then every chunk submitted to the Up Tree competition gets a fraction of time in STM proportional to its importance as determined by f. Specifically, the probability that a submitted chunk_p,t,0_ gets into STM is^‡‡^

$$f\left(c\mathrm{hun}k_{p,t,0}\right)/\sum_{\text{all}\;N\mathrm{LTM}\;\text{processors}\;p’}f\left(c\mathrm{hun}k_{p’,t,0}\right).$$

As a consequence, for additive f, the permutation chosen to assign processors to leaves of the Up Tree has no effect on the sequence of broadcasts from STM. Additionally, even those chunks with low f value have some chance of getting into STM. (Ref. 48 has more specifics, including the statements of this and other theorems and their proofs.)^§§^

### 2.4. Complexity of Computation and Time Delay for Conscious Awareness.

For t > 0 and s > 0, the computation to update the chunk at node v_s_ in the Up Tree competition consists of

1) two fast computations of f, a sum and division of their values, and a fast probabilistic selection;
2) putting the address, gist, and weight of the chunk selected into v_s_; and
3) summing the intensities and moods of the chunks associated with v_s_’s children and setting those sums to be the intensity and mood of the chunk at v_s_.

These computations, all three of which must be completed in 1 time unit, put a bound on both the size of the chunk in a node and the amount of computation that can be performed in that node.^¶¶^

### 2.5. Memories and the High-Level Story.

We assume that each processor p stores in its internal memory the sequence of tuples, ordered by time t, consisting of the chunk_p,t,0_ that it submitted to the competition, the chunk it received at time t by broadcast from STM, and a select subset^##^ of chunks it received at time t from links or from input maps. These sequences are a substantial part of CTM’s **memories**.

This “history” provides a **high-level story** of what p saw and did. High-level stories account in large part for CTM’s sense of self in its feeling of consciousness (section 3). CTM calls on high-level stories coupled with prediction algorithms to create dreams (section 4.5).

Periodically, this stored information may be pruned so only “salient” chunks remain, the most salient being those that represent terrible, wonderful, or unexpected events. In general (section 2.6), every processor makes predictions regarding the chunks it generates, modifies, and stores.

### 2.6. Predictive Dynamics = Prediction + Feedback + Learning (Sleeping Experts Algorithm).

Processors require feedback to assess correctness and detect errors in their predictions and to learn how to both boost correctness and diminish and correct errors.

• **Predictions** in CTM are made by LTM processors for all chunks whether submitted to the competition for STM, to other processors through links, or to actuators that effect the environment.
• **Feedback** comes from chunks that are received in broadcasts from STM, through links, and from the environment via input maps.
• All CTM **learning** and **error correcting** take place in processors.

There is a continuous cycling of prediction, feedback, and learning within CTM. The CTM needs to be alerted to anything unusual, surprises of any kind, in order to deal with such things if necessary and to improve its understanding of the world in any case. Prediction errors (e.g., “surprises”) are minimized by this cycling.

Processors especially need to know if they were too timid or too bold in setting their |weights|, so they can correct their weight-assigning algorithms. **Sleeping Experts Algorithms** (SEAs) are a class of learning algorithms employed by LTM processors to do just that [Blum and coworkers (59–61)]. Here is one of the simplest versions of the SEAs.

Embolden a processor (raise the intensity it gives its chunks) if

1) its chunk did not get into STM and
2) its information is more valuable (in the SEA’s opinion) than what got into STM.

Hush a processor (lower the intensity it gives its chunks) if

1) its chunk got into STM and
2) its information is found (perhaps later) to be less valuable than that of some chunk that failed to get into STM.

SEAs play a role in whether or not processors get their chunks into STM. They also play a role in whether or not processors “pay attention” to gists in chunks that are sent to them via links. The |weight| of a chunk is an indication of how important the processor that generated the chunk believes its gist to be, and this will influence whether or not a processor that receives the chunk will pay attention to it.

### 2.7. Comparison of CTM with the GWT Model.

**Fig. 2.**
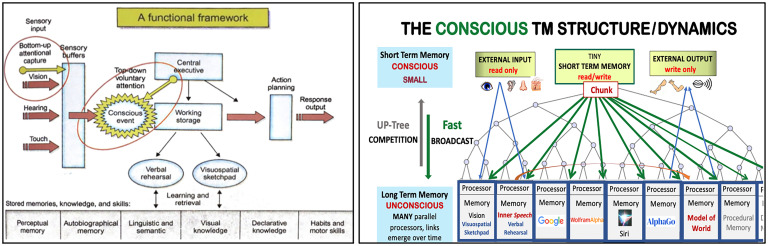
Sketches of models: Baars' GWT model (left) and the CTM (right).

We conclude this section with a comparison between the CTM and Baars’ GWT model (Fig. 2).

Aiming for simplicity, we have eliminated or simplified many features. For example, the CTM has just one “actor” on stage holding just one chunk at a time. Additionally, all processors in the CTM are in LTM. We have eliminated the central executive since its functions can be handled by processors. In the CTM, inputs and outputs go directly to and from LTM processors, not directly through STM.

In the CTM, chunks compete in a well-defined competition to get onto the stage (STM). Conscious awareness (attention) is the reception by all LTM processors of the broadcasted winning chunk (i.e., CTM’s conscious content), not an event that occurs between input and STM. The roles of Baddeley and Hitch’s verbal rehearsal and visuospatial sketchpads (62) are assumed by LTM processors.

**Predictive dynamics** (cycles of prediction, feedback, and learning) and a multimodal inner language (Brainish) as well as computational and complexity considerations are explicit key CTM features.

Finally, as in the “extended mind theory” (63), CTM can have access to existing technology, such as Google, Wikipedia, WolframAlpha, AlphaGo, and so on, in the form of LTM processors tasked to use these apps. This is one way to ensure that CTM has a huge collection of powerful processors at the start of its life (t = 0), a collection that is augmentable throughout its life.

Key features of the CTM model and its dynamics resonate with properties of consciousness that Dennett (64) outlines.

*[Neither] a Master Scheduler, nor a Boss Neuron, nor a Homunculus or Res Cogitans [govern the transitions of our conscious minds]. [What governs] must be a dynamical, somewhat competitive process of contents vying for fame, for cerebral celebrity … or relative clout against the competition. What determines the winners? Something like micro-emotions, the strength of positive and negative valences that accompany and control the destiny of all contents, not just obviously emotionally salient events such as obsessive memories of suffering or embarrassment or lust, but the most esoteric and abstract theoretical reflections.*

Although inspired by Baars’ GWT architecture, the CTM integrates features essential for its feeling of consciousness. This is the focus of the next section.

## 3. The Feeling of Consciousness

While CTM is **consciously aware** by definition of the conscious content broadcast from STM (section 2.1.3), this definition does not explain what generates the feeling of consciousness in CTM.

We argue that the feeling of consciousness in CTM is a consequence principally of its extraordinarily expressive Brainish language, coupled with CTM’s architecture, certain special processors, and CTM’s predictive dynamics (prediction, feedback, and learning).

1) **Brainish**. The multimodal Brainish language describes the sensory world exactly as it is perceived. This perception consists of gists in the multimodal language of sensations. Its words include gists for odors (the odors as they are perceived by the nostrils), pains (the terribly unpleasant sensations of pain), faces (what one sees when looking at someone’s face), and so on. Dreams are important because they show what gists can express when the CTM has neither input nor output.
2) **Architecture**. This includes the Up Tree competition to gain access to STM (section 2.3) and subsequent global Down Tree broadcast of the winner to all LTM processors (particularly all processors that play a special role in generating the feeling of consciousness.
3) **Special processors**. We single out a few such processors, which have specialized algorithms built into them at birth.
  a) The **Model of the World** processor constructs models of CTM’s worlds based on information it gets from the environment or from stored possibly modified inner memories. We define CTM’s **inner world** to be the sparse “CTM” model that the Model of the World processor creates of the CTM. We define CTM’s **outer world** to be the model it creates of the environment. Importantly, the Model of the World processor tags parts in its models of the world (inner and outer) with labels and descriptions annotated in Brainish with sensations they (can) have and actions they (can) perform.
  b) The **Inner Speech** processor extracts whatever speech is encoded in the gist broadcast by STM and sends it to the same locations that the input map sends gists of **outer speech** (the gists created by the input maps). This is sent initially via STM and then later after links are formed, via links directly. **Inner speech**, the gists produced by the Inner Speech processor, enables CTM to recollect its past, predict its future, and make plans. The gists of inner speech *(such as those that occur in talking to oneself or the talking and hearing in a dream)* are nearly indistinguishable from the gists of outer speech. *In humans, inner speech sounds so much like outer speech that it can be difficult, as in schizophrenia, to distinguish between inner and outer speech (65).*
  c) **Inner Vision** and **Inner** (tactile) **Sensation** processors map whatever images and sensations are broadcast from STM to whatever locations the input maps send outer scenes and outer sensations. The gists of **inner vision** can be barely distinguishable from the gists of **outer vision** (the visual gists created by the input maps). CTM’s memories and predictive abilities enable CTM to create the **inner images** and **sensations** that CTM uses to generate imaginings and dreams (section 4.5). *To thwart schizophrenic hallucinations, the human brain distinguishes inner images from outer images. The brain has various tricks for doing this, one being to make dreams hard to remember.*

These processors inform the “eyes” and “skin” in CTM's models of the world that “see” whatever the CTM recalls from visual memory and “tactily sense” whatever CTM recalls from sensory memory. These eyes and skin are CTM’s mind’s eye and mind’s skin. We consider these processors to be Inner generalized Speech processors.

4) **Predictive dynamics**. Additionally, we argue that CTM’s continuous cycling through prediction, feedback, and learning (section 2.6) plays a role in CTM’s feelings of consciousness (1). The feelings are further enhanced by (parallel) predictive dynamics in CTM’s Model of the World processor, where planning and testing are constantly carried out by the CTM. Positive feedback gives CTM an indication that it understands what is going on; negative feedback—unless it is about something that could not have been predicted, such as an unexpectedly loud noise—gives CTM evidence of something that it did not know or understand.

We also add

5) a minimal (general) ability to think and make plans and
6) the motivation (= energy + drive) to make a plan and then pursue it.

We now return to the Model of the World processor to describe a central task, that of tagging various constituent parts of its models as either self or not self (else unknown).

How does the Model of the World processor determine what is or is not self? If the broadcast of a chunk (a CTM thought) is immediately followed by an actuator carrying out an action in the environment—and that same thought leads to the same action consistently and repeatedly—then that indicates the actuator is part of self. The Model of the World processor has additional important jobs that give the CTM its sense of self, including creating imaginings, creating maps of its environment and registering movements in its environment, helping to plan actions in the environment, helping to predict the actions of self and not self in the environment, and correcting predicted actions of self and not self.

When (through broadcasts) the CTM detects itself thinking about its own consciousness, the Model of the World processor tags the "CTM" in its models as “conscious.”

We now look at why the CTM considers itself conscious. It cannot be because the Model of the World processor or any other processor feels it is conscious, as processors are just machines running algorithms—and (such) machines have no feelings. We propose that CTM as a whole feels conscious, as the term is normally understood, as a consequence in part of the fact that the Model of the World processor views the "CTM" in its models of the world as conscious and that this view is broadcast to all processors. Here, "CTM" is a simple learned representation of the much more complex CTM. Our explanation for CTM’s feeling of consciousness aligns closely with the attention schema theory of Michael Graziano et al. (18).

## 4. High-Level Explanations

We now explore how CTM might experience a variety of phenomena generally associated with consciousness. We believe that our explanations, derived from the model, provide a high-level understanding of how conscious experiences are or might be generated. These draw confirmation from consistencies with the psychology and neuroscience literature at a high level.

Previously (48), we explored explanations for the feelings of pain and pleasure in the CTM. Here, we consider additional phenomena, again from the perspective of the CTM. We start with three examples related to vision (blindsight, inattentional blindness, and change blindness) and then follow with a discussion of dreams and free will.

### 4.1. Blindsight.

Blindsight provides a striking example of the difference between conscious and unconscious awareness (66). *In blindsight, the person does not consciously see the outer world. When asked to fetch something across a cluttered room, a typical response is “but I cannot see.” Nevertheless, the person can respond adeptly if cautiously to the request.* What is going on?

In the CTM, visual input goes directly from the vision sensors to a subset of LTM processors that process visual input. However, in the blindsighted CTM due to some malfunction, perhaps a break in the Up Tree or some other inability for the vision processors to enter chunks competitively into the competition, this information does not get up to STM and hence, does not get globally broadcast. For this reason, CTM is not consciously aware that it can see. However, information can still be communicated between (unconscious) processors via links. So, visual information received by the vision processors can be sent through links to the walk processor that controls the leg actuators. *At a high level, this explanation is consistent with explanations of blindsight in humans given in ref. 67.*

### 4.2. Inattentional Blindness.

Inattentional blindness occurs when an individual fails to perceive a visual stimulus that is in plain sight. It is “the failure to notice the existence of something unexpected when attention is focused on some other task” (68).

*For example, in the famous selective attention test (69, 70), viewers of the “invisible gorilla” film were asked to “count how many times the players wearing white shirts pass the basketball.” Nearly all viewers gave close to the correct number (48) but were stunned when asked, “Did you see the gorilla?”* What is going on?

Let us suppose the CTM is viewing the gorilla film. The input query about the white-shirted players gains access to STM and is then immediately broadcast to all LTM processors. To carry out the task, CTM’s vision processor assigns high intensities to white-shirted gists and very low intensities to anything black. The chunk with the “gorilla” gist has little chance to enter STM. The CTM does not consciously see the gorilla.

The CTM explanation of inattentional blindness reduces to the differential intensities given to gists, lower intensities given to irrelevant ones, and the competing advantages of chunks with higher intensities.

*According to simulations performed in ref. 10, during certain “ignited” states, “spontaneous activity can block external sensory processing.” They relate this blocking to the cause of inattentional blindness.* In our view, blocking the “sensory processing” in human brains of black objects is roughly equivalent to the CTM dramatically lowering the intensity of black gists in chunks, thus lowering the chances of those chunks entering STM. The effect of differential intensities in the CTM is also consistent with theoretical implications *that inattentional blindness in humans “can serve as a filter for irrelevant information. It may also filter out unexpected events” (68).*

### 4.3. Change Blindness.

Change blindness occurs when individuals fail to notice large changes in pictures or scenes (71). It is “the failure to notice when something has changed from one moment to another” (68).

*An instructive example is the Whodunnit video (72). A detective enters a murder scene proclaiming, “Clearly somebody in this room murdered Lord Smythe” and immediately interrogates each suspect in turn. The maid proclaims, “I was polishing the brass in the master bedroom.” The butler says, “I was buttering his Lordships scones,” and Lady Smythe says, “I was planting my petunias in the potting shed” (enough information for the clever detective to solve the murder on the spot).*

However, why did we not notice the many incongruous scene morphs between the beginning screen shot and the end?

From the perspective of the CTM, in viewing the Whodunnit video, CTM has the impression of seeing the whole but does not notice the changes that take place as the trench coat, flowers, painting, and so on are replaced by variants. That is because of the following.

1) The filming is cleverly staged so that there are cuts from the whole scene to the suspects (e.g., the maid alone), eliminating transitions that show the dark trench coat replaced by the white one, the bear replaced by the suit of armor, the rolling pin replaced by the candelabra, the dead man now with a change of clothes and raised leg, and so on. The video input never signals CTM’s vision processor that the “scene” has been modified.
2) Importantly, the same gist describes both the beginning and ending scenes equally well: “The living room of a mansion with detective, butler, maid, others, and a man apparently dead on the floor.”

Under these conditions, the CTM experiences change blindness.

Again, the CTM explanation is consistent with the literature on change blindness in humans. *For example, according to ref. 68 confirming earlier work in ref. 71*,

*[g]iven that change detection requires adequate representation of the pre- and post-change scenes as well as a comparison, any task characteristics that influence the richness of the representation or the tendency to compare representations should affect detection. The semantic importance of the changing object appears to have the biggest influence on the likelihood the subject will attend, and therefore notice, the change.*

### 4.4. Illusions.

Inattentional blindness and change blindness might be considered examples of illusions.

The CTM is consciously aware by definition of the gists (in chunks) that are broadcast from STM. (Those gists reached STM from LTM. LTM got them directly from sensors via input maps, from other LTM processors through links, and from STM by broadcasts.) The gists are stored in LTM memories for many reasons, one being to supply the processors’ high-level stories (section 2.5), such as those that occur in dreams.

In CTM, the stream of consciousness is the sequence of gists broadcast from STM (section 2.1.3). Each visual gist at each moment gives the CTM the sense that it sees the entire scene before its eyes, although it sees at most a tiny fraction of the scene. The illusion of the whole has several explanations, the main one being that a multimodal Brainish gist can describe a hugely complex scene, like “I’m standing before a Japanese style garden containing a brook, path, bridge, and trees.” Could that gist contain the details of a 12-million-pixel photograph from an iPhone camera, which is what it feels like we are seeing? The illusion of the whole is a consequence of the highly suggestive (succinct) information in a gist. The CTM conjures up the scene in a kind of magic act. Keith Frankish (73) calls this the illusionism theory of consciousness.

### 4.5. Dream Creation.

Dreams are the ultimate illusions. *Some people claim not to dream, but most do (74). Their dreams may be visual, auditory, tactile, etc. They are often related to emotional processes (2, 75). They can express great pain and fear (nightmares) or great pleasure (as in flying dreams). One can feel crippling pain in the leg and wake up to find that the pain is completely illusory; there is no pain at all. One can be lying face down and wake face up*.

In the CTM, a built-in **Sleep** **processor** keeps track of time, habits, day/night, etc. and has internal algorithms to monitor the need for sleep. If and when the Sleep processor determines that sleep is needed, it takes control by raising the intensity of its own chunks enough to get them into STM and to keep other chunks out. This has roughly the same effect as lowering the intensities of chunks from other LTM processors. It also blocks or greatly reduces the intensity of various inputs (eyes and ears), and it blocks signals that activate outputs (such as to limbs). The CTM sleeps. This is the **sleep state**.

The Sleep processor continuously monitors the need for sleep and as that need diminishes, reduces the intensity of its own chunks proportionately. This eventually permits **dream gists** (in chunks) to reach STM. This is the **dream state**. Finally, when the Sleep processor releases its choke hold on inputs and outputs, the CTM **wakes up**. That is in the CTM. *In humans, non-REM sleep and REM sleep can alternate several times before awakening (76).*

When CTM is in the dream state, a processor acting as **Dream** **Creator** becomes active (that is, starts getting its chunks into STM). The gists in these chunks contain kernels of ideas (typically based on earlier CTM activities, concerns, imaginations). When these chunks are broadcast, all processors, including those that play key roles in the feeling of consciousness, receive those broadcasts and compete to respond. This gives the CTM the same sense of being alive while in the dream state as when it is awake.

The Dream Creator and the other processors take turns interacting back and forth. The conversation—the back-and-forth interaction—between Dream Creator and the gamut of processors is the sequence of gists that constitutes the **dream**. This sequence is the **dream stream of consciousness**.

The dream essentially stitches together this sequence of chunks to produce a dream stream of consciousness (inner movie) that 1) sees, hears, and senses the dream world and 2) affects what appears in that dream world. Such an (interactive) inner movie displays a range of sensory inputs (images, smells, and sounds) and generates a range of actions.

When the CTM is asleep but not dreaming, most processors cannot get their chunks into STM. Exceptions include detectors of especially loud noises and the sleep processor itself. The Sleep processor’s chunk in STM blocks most other processors’ chunks from reaching STM. By design, it holds an empty gist, so the CTM is not conscious or barely so.

After the CTM leaves the sleep state to enter the dream state, a fraction of LTM processors, such as the Inner Vision processor, can get their chunks into STM. Thus, while dreaming, the CTM is conscious and can experience events vividly.

As discussed earlier (section 3), key processors, such as those for Inner Speech, inner Vision, Inner Sensations, and Model of the World, play special roles in generating the feeling of consciousness in CTM. These processors play similar roles when CTM is dreaming.

Here are some examples of how processors help with dream creation.

• The Inner Speech processor culls the inner speech from the multimodal gists broadcast from STM and sends that speech to the same processor that receives outer speech. This process causes speech in dreams to sound like outer speech. The Inner Vision and Inner Sensation processors help in a similar way with dream creation.

Dreams demonstrate the power of Brainish gists. What CTM sees, hears, feels, and does in a dream is necessarily fabrications by processors that can recall, modify, and submit creations to the competition for STM. These fabrications are realistic because they use the same gists that are generated while awake. Thus, dreams generate the sense of a realistic world even while CTM is completely divorced from external inputs. As a consequence, they can appear so realistic that for CTM, *as for humans (77)*, it may become hard to distinguish dreams from reality. *(This problem is avoided in humans if dreams are hard to remember.)*

High level confirmation comes from research [ref. (78) referring to (79)], *demonstrating* *that* *in humans, the same neural pattern of activity occurs when one sees a face, brings the face back from memory, or when the face appears in a dream. They also point out that in REM sleep, the activation of the motor cortex in a dream, when one has the sensation of movement, is the same activation as when awake.*

• The Model of the World processor predicts the effect that CTM’s actions will have in its (inner and outer) world. It does this from the effect of those actions in its models of the world. The Dream Creator can use this same prediction machinery to create dreams.

Dreams also enable the CTM to test itself in unknown and possibly dangerous situations. In both humans and CTM, dreams can be laboratories for experimenting with various possible solutions.

However, unlike what occurs in waking consciousness, inconsistencies are more likely to occur unnoticed in dreams than while awake since the CTM’s “consistency checkers” in its Model of the World processor are not getting input from the environment. Hence, the CTM can fly in its dreams.

Zadra and Stickgold (78) assert that* in humans, “Dreams don’t replay memories exactly; they create a narrative that has the same gist as some recent memory and could have the same title.” They note that “REM sleep provides a brain state in which weak and unexpected associations are more strongly activated than normal strong associations, explaining how it aids in finding the remote associates and perhaps explaining the bizarreness in our REM sleep dreams.”*

### 4.6. Free Will.

The problem of free will is ancient. It appears in Lucretius (*De Rerum Natura*, first century BC): “If all movement is always interconnected, the new arising from the old in a determinate order—if the atoms never swerve so as to originate some new movement that will snap the bonds of fate, the everlasting sequence of cause and effect—what is the source of the free will possessed by living things throughout the earth?” (80).

The paradox of free will is captured by Dr. Samuel Johnson’s (1709 to 1784) observation (81): “All theory is against the freedom of the will; all experience is for it.”

Stanislas Dehaene (15) bestows a contemporary voice: *“Our brain states are clearly not uncaused and do not escape the laws of physics—nothing does. But our decisions are genuinely free whenever they are based on a conscious deliberation that proceeds autonomously, without any impediment, carefully weighing the pros and cons before committing to a course of action. When this occurs, we are correct in speaking of a voluntary decision—even if it is, of course, ultimately caused by our genes [and circumstances].”*

We add to Dehaene that computation takes time. To make a decision, CTM evaluates its alternatives in an evaluation that takes time, and during that time, the CTM is free, indeed can feel free, to choose whichever outcome it deems (computes) best.

The TCS perspective thus informs our definition of free will.

Free will is the freedom to compute the consequences of different courses of action—or as much of those consequences as is possible within the available resources (time, space, computational power, and information)—and to choose from them whichever course of action best suits one’s goals.

This definition incorporates both predictive dynamics (compute the consequences of different courses of action) and resource constraints (time, space, computational power, and information).

For example, consider a CTM that is called on to play a given position in a game of chess. Different processors suggest different moves. The CTM’s main chess-playing processor (assuming one exists; otherwise, a processor that has a “hi-level” view of the game) indicates, by broadcast of a chunk in STM, that it recognizes it has a choice of possible moves and that the decision of which move to make merits a careful look at the consequences of each move. At this point, faced with a selection of possible moves but not yet having evaluated the consequences of those moves, the CTM is free to choose whichever move it reckons best within the time constraints.

Will the CTM feel that it has free will?

1) Consider the moment that the CTM asks itself “What move should I make?,” meaning that this question has risen to the STM stage and through broadcast, has reached the audience of LTM processors. In response, a number of those processors submit suggestions to the competition. The winner of the competition reaches the stage and gets broadcast. Because gists are short, any such broadcast is short and therefore, reasonably articulable.
2) The continued back-and-forth comments, commands, questions, suggestions, and answers that appear in STM and globally broadcast to LTM give the CTM a knowledge of its control. If the CTM was asked how it generated a specific suggestion (i.e., what thinking went into making that suggestion), its processors would be able to articulate the fraction of conversation that reached the stage (although perhaps not much more than that in the short term).
3) Many LTM processors compete to produce the CTM’s final decision, but CTM is only consciously aware of what got into STM, which is not all of what was submitted to the competition. Moreover, much of CTM, meaning most of its processors, is not privy to the unconscious chatter (through links) among processors. To the CTM, enough is consciously unknown about the process that the decision appears at times to be plucked from thin air. Even so, although CTM does not consciously know how its suggestions were arrived at, except for what is in the high-level broad strokes broadcast by STM, it knows that its suggestions came from inside itself. The CTM can rightly take credit for making its suggestions (after all, they did come from inside the CTM) and can explain some of them with high-level stories (section 2.5), and as for what it cannot explain, it can say “I don’t know” or “I don’t remember.” It is the knowledge that there are choices—that it (the CTM) has knowledge of and about those choices and that it has ignorance as well—that generates the feeling of free will. Deterministic or not, the experiential feeling is one of free will.

How important is randomness for this explanation of the feeling of free will? Notice that no quantum physics is required in the CTM for the above explanation. The only randomness is that of the coin-flip neurons in the Up Tree competition and whatever randomness, if any, the processors use in their probabilistic algorithms. It can be shown, moreover, that the above argument for the feeling of free will still applies for a completely deterministic CTM (e.g., one that uses pseudorandomness). It follows—and we expect this will be a source of contention—that even in a completely deterministic world, the CTM will feel it has free will.

## 5. Summary of Methods

We consider consciousness from the perspective of TCS, a nonexperimental area of mathematics. Inspired by Alan Turing’s simple yet powerful model of a computer, the TM, and by Bernard Baars’ *Theater of Consciousness*, we define a substrate independent computational model of consciousness, the CTM. Formal definitions of conscious content, conscious awareness, and the stream of consciousness in the CTM are followed by arguments that the CTM supports high-level explanations for these and other phenomena associated with consciousness, including the feeling of consciousness. One purpose of our model is to argue these claims. Another is to provide a TCS foundation for understanding consciousness.

*SI Appendix* and ref. 49 extend this paper with additional figures and frequently asked questions (FAQ).

## Supplementary Material

Supplementary File

## Data Availability

This is a TCS paper. There are no data underlying this work, but please see the linked *SI Appendix* for additional information including an extended summary, a new section on altered states, and relations of the CTM to other theories of consciousness.

## Footnotes

*The various approaches to the study of consciousness include psychological (1–3), philosophical (4–7), information theoretic measures (8, 9), neural correlates (10–12), and evolutionary (13). Our approach to consciousness is architectural, close in spirit to refs. 14–17. It is also close in spirit to refs. 18–20. Related approaches include predictive processing (21–26). The architectural approach to the study of consciousness was inspired by the architectural models of cognition. These were developed largely at Carnegie Mellon University in Herb Simon’s *The Sciences of the Artificial* (27), Raj Reddy’s Blackboard Model (28), Allen Newell’s *Unified Theories of Cognition* (29), and John Anderson’s ACT-R (30). The global workspace idea is due to Newell (29). An important more recent architectural model of cognition is LIDA (31).

^†^Although a TM looks nothing like a modern-day computer, Turing did intend to construct a practical programmable computing machine. In 1945, he brought with him to the British National Physical Laboratory an 86-page detailed blueprint for a universal programable computer, the automatic computing engine (ACE), which he intended to build (33). Politics intervened, and only the less ambitious Pilot ACE (34) got built.

^‡^Before Gödel and Turing, mathematicians had the unshakable belief that with enough knowledge and work, any mathematical problem could be solved. As the mathematician David Hilbert famously said in 1930 at his retirement address: “This conviction of the solvability of every mathematical problem is a powerful incentive to the worker. We hear within us the perpetual call: There is the problem. Seek its solution. You can find it by pure reason, for in mathematics there is no ignorabimus [sic]” (37).

^§^Suppose that when program P is run on computer C on any input of length n, it has run time 2^n^. Let C^+^ be the same computer except that C^+^ runs twice as fast as C. Then, program P, run on computer C^+^, on any input of length n + 1 will have run time 2^n+1^/2 = 2^n^, which is the same running time as C on any input of length n. TCS considers the increased speed of C^+^ (and therefore, C^++^, C^+++^, …) over that of C to be insignificant for running P when n is large.

^¶^Solvable in polynomial time (P) means it is possible to find a solution in time polynomial in the size of the problem instance. Checkable in polynomial time (NP) means that given a purported solution, its correctness can be checked in time that is polynomial in the size of the problem instance. On the face of it, finding a solution seems harder than checking it; properly coloring the nodes of a graph with three colors is hard (NP hard so likely not in P), while checking if a three coloring is proper, meaning that no edge joins two nodes of the same color, is easy (in P).

^#^We note a historical synergy between TCS and neuroscience. Turing’s simple computer model led neuroscientist Warren S. McCulloch and mathematician Walter Pitts (50) to define their formal neuron, itself a simple model of a neuron. Mathematics forced their model to have inhibition, not just excitation—because without inhibition, loop-free circuits of formal neurons can only compute monotonic functions—and these do not suffice to build a universal TM. The McCulloch–Pitts neuron also gave rise to the mathematical formalization of neural nets (51) and subsequent deep learning algorithms (52), further illustrating ongoing synergies.

^ǁ^*In humans, the storage capacity of STM is roughly 7 ± 2 chunks (54), where a chunk can be a word, a phrase, a digit, and so on.* A few chunks cycling through STM can simulate some aspects of an STM that holds several chunks. Cycling can happen via the Up Tree competition and the Down Tree broadcasts. In this way, CTM can keep thoughts alive in STM continuously through many cycles by sending the thought from processor to STM to processors to STM and so forth.

**A primary reason to keep the number of chunks in STM small (exactly one in our model) is to ensure that all processors focus on the same information in the broadcast from STM. (Another reason is to keep the model as simple as reasonably possible for an understanding of consciousness.) Leslie Valiant (ref. 55, pp. 127–128) does not assert that focus is primary. He asserts instead that limited computational resources and constraints imposed by the need to learn are the primary reasons for the small size of conscious information. While these are reasonable factors, the principle factor in our opinion is the required focus.

^††^If the CTM has 10^7^ LTM processors, *corresponding to ≈10^7^ cortical columns in the brain*, and if all pairs of processors are initially linked, there will be ≈10^14^ links, which is a large possibly infeasible number of links. Worse yet, a processor with 10^7^ inputs might need to run its own personal competition to decide what to look at. Although one might want certain special processors linked initially, we choose for simplicity to have no processors linked at birth.

^‡‡^The following questions arise. Why is a tree necessary? Why not just compute f(chunk_p,t,0_)/∑_all N LTM processors p’_ f(chunk_p’,t,0_) in one step to get the probability of a chunk winning the competition? The answer is that, however you do it, you need log **N** steps to compute the ∑.

^§§^We note that f(chunk_p,t,s_) = **|**mood_p,t,s_**|** is a bad choice for more reasons than that it is not additive. If at level 0, two sibling chunks have weights +100 and −100 and all other chunks have weight = +1, then neither of the two high-intensity siblings will reach STM. *This might occur if you spy a $10 bill on the ground, but someone else picks it up. In that case, the pleasure of finding the $10 bill and the pain of losing it will never reach consciousness. You would be unconscious of both. This seems unlikely to us*.

^¶¶^The space required to store a chunk must be large enough to store a log_2_N bit address and to store a gist whose length is no greater than what is required to store approximately one line of English or its equivalent in Brainish, very roughly 2^10^ bits. If 1 time unit is 100 ms and there are 10^7^ LTM processors, then the time from a chunk being placed into competition by an (unconscious) LTM processor to becoming CTM’s conscious content will be about 2.3 s. If the broadcast takes another 100 ms, the total time to conscious awareness will be about 2.4 s. This time can be reduced from 2.4 to 0.7 s if the interior nodes of the Up Tree each have 10 children instead of 2. (Section 4.6 has the implications of an Up Tree delay on free will in the CTM and/or humans.)

^##^ *Assuming that CTM is a good model for the brain, it is not possible for each of its **N** ≈ 10^7^ processors to record from all other **N** − 1 processors, as that would require CTM to have more links than there are neurons in the brain.*

## Change History

May 25, 2022: The SI Appendix has been updated.
