# Supplementary material for: A theory of consciousness from a theoretical computer science perspective: Insights from the Conscious Turing Machine
Source: Proc Natl Acad Sci U S A. 2022 May 20;119(21):e2115934119. doi: 10.1073/pnas.2115934119 (PMC9171770; doi:10.1073/pnas.2115934119)
Supplement: Supplementary File [file pnas.2115934119.sapp.pdf]

A Theory of Consciousness from a Theoretical Computer Science Perspective: Insights from the Conscious Turing Machine

Lenore Blum[*1] **and Manuel Blum**[**2]

Appendix

This **Appendix** contains the following sections: **Extended Summary**, **FAQ**, **Altered States (new section)**, **Relations of CTM to Other Theories of Consciousness**, **About the Authors**, **Figures** and **References**.

Please also see: https://arxiv.org/pdf/2107.13704.pdf (Blum & Blum, 2022)

Key words: consciousness, theoretical computer science, substrate independent model, computational model, global workspace, multi-modal, world models, phenomenal consciousness, the hard problem.

In what follows, statements about the Conscious Turing Machine (**CTM**) are printed in black. Statements particular to humans or animals are generally printed in burgundy.

Table of Contents

1 **EXTENDED SUMMARY** 2

2 **FAQ** 3

3 **ALTERED STATES OF CONSCIOUSNESS (ADDED SECTION)** 8

4 **RELATION OF CTM TO OTHER THEORIES OF CONSCIOUSNESS** 9

5 **ABOUT THE AUTHORS OF THE EXPANDED MONOGRAPH** 11

6 **FIGURES** 12

7 **REFERENCES** 14

[*] Lenore Blum, Emeritus, Computer Science Department, Carnegie Mellon University, Pittsburgh, PA 15213; EECS, UC Berkeley, CA 94720; 700 Euclid Ave, Berkeley CA 94708, (412) 414-8730, lblum@cs.cmu.edu; https://orcid.org/0000-0003-1541-1294

[1] Equal contribution.

[**] Manuel Blum, Emeritus, Computer Science Department, Carnegie Mellon University, Pittsburgh, PA 15213; Emeritus, EECS, UC Berkeley, CA 94720; 4770 Bayard St, Pittsburgh PA 15213, (412) 596-4063, mblum@cs.cmu.edu; https://orcid.org/0000-0002-0982-4845

[2] Equal contribution.

© 2022 Blum & Blum

1 Extended Summary

We consider consciousness from the perspective of theoretical computer science (**TCS**). Inspired by Alan Turing's simple yet powerful model of a computer, the Turing Machine (**TM**), and by Bernard Baars' *Theater of Consciousness,* we define a computational model of consciousness, the **Conscious Turing Machine** (**CTM**).

The **CTM** is *defined formally* as a **7**-tuple, **< STM, LTM, Up-Tree, Down-Tree, Links, Input, Output >**. The theory includes a precise definition of George Miller's informally defined **chunk**, and a precise definition of a **competition** for deciding which of the (10^7 or more) **Long Term Memory (LTM)** processors gets access to **Short Term Memory (STM)**.

Bi-directional **links** between processors that *emerge* in the life of the **CTM** enable conscious processing to become unconscious. Links are also crucial for the "global ignitions", described by (Dehaene & Changeux, 2005) in their **Global Neuronal Workspace Theory (GNWT)**, that re-enforce and sustain conscious awareness. **Input/Output** maps enable communication between the **CTM** and its **environment**. Other features of the model can be found in (Blum & Blum, 2021).

The definition of the model is followed by formal definitions of **conscious content**, **conscious awareness**, and the **stream of consciousness**, in the **CTM.** While these are just formal definitions, we claim that the **CTM** supports *high-level* explanations for these and other phenomena associated with consciousness, including the *feeling* of consciousness. One purpose of our model is to argue these claims. Another is to provide a theoretical computer science foundation for understanding consciousness.

In particular, we argue that the *feeling* of consciousness arises in **CTM** as a consequence of:

1. the global workspace *architecture,* which enables all processors, including those that are particularly responsible for the feeling of consciousness - **Inner Speech**, **Inner Vision**, **Inner Sensations** and **Model-of-the-World** – to be privy to the same (conscious) content of **STM**,
2. the *expressive power* of **CTM**'s multi-modal inner language *Brainish*, which is able to express gists that betoken images, sounds, tactile sensations, thoughts, pains, pleasures, and the whole range of emotions,
3. the close correspondence between gists of outer speech (what we say and hear in the world), outer vision (what we see in the world), and so on, to gists of inner speech (what we say to ourselves), inner vision (what we see in dreams), and the like, and
4. *predictive dynamics* = cycles of prediction, feedback, and learning that help **CTM** develop its understanding and its ability to deal with its environment and inner world.

We argue that the *feeling* of free will in the **CTM**, like the *experiences* of illusions and dreams, are direct consequences of **CTM**'s *architecture,* certain *special processors* such as the **Model-of-the-World** processor and the **Inner generalized Speech** processors, the expressive power of **Brainish**, and its *predictive dynamics*.

© 2022 Blum & Blum

2 FAQ

Q1. What is the advantage of having the CTM architecture?

A. When a processor doesn't know where to find the information it needs, a global broadcast requesting that information can get other processors engaged in finding it. That's one example. Multi-modal integration of information is another functionality achieved by this architecture.

Q2. Why does the STM hold only one chunk?

A. George Miller suggested the magic number 7±2 for the number of chunks in human short-term memory (Miller, 1956). Having a small number such as this is important for focusing attention. Some folks are incredulous that so few chunks will suffice. The **CTM** emphasizes this point by having just 1 chunk.

Q3. Why is the CTM defined in the specific format given (and not some other)?

A. The CTM is defined by the 7-tuple **< STM, LTM, Up-Tree, Down-Tree, Links, Input, Output >**. Other formats may be just as good. Some format had to be chosen. Turing chose sets of quadruples for his machines (Turing A. M., 1937).

Q4. What distinguishes conscious processing from unconscious processing?

A. Conscious processing is what gets broadcast from **STM** plus whatever communications about that broadcast through links between processors continue to keep it alive, what (Dehaene & Changeux, 2005) call "ignition". Unconscious processing is all the rest. Processing that does not reach **STM** does not get broadcast and therefore remains unconscious.

Q5. Why is the Up-Tree a strictly binary tree?

A. The binary **Up-Tree** could more generally be a **k**-ary tree for some small **k**, **k** much less than **N** (the number of processors). The original Turing Machine (**TM**) had just one read/write head and one 1-dimensional tape. Since then, others have considered **TM**'s with multiple tapes, multiple heads per tape, multi-dimensional tapes, and so on. In a similar way, the **Up-Tree** is made binary because binary is both *simple* and *sufficient*, and because the choice between 2 chunks at a node is slightly simpler to describe.

Q6. Why an Up-Tree at all?

A. We need a vehicle for **LTM** processors to get **CTM** to pay attention to the "most important" information. We chose the **Up-Tree** to execute the competition in part because it computes locally (between 2 siblings) to get the globally most important information into **STM.** With an additive competition function, the **Up-Tree** structure and competition ensure that processors get their chunks into **STM** with probability proportional to their **f**-value, which is arguably the correct way to do it. We consider the **Up-Tree** and the decisions it makes to be of fundamental importance.

The question also arises: Why not just compute $\mathbf{f(chunk_{p,t,0}) / \sum_{all\ N\ LTM\ processors\ p'} f(chunk_{p',t,0})}$ in one step to get the probability of a chunk winning the competition? Answer: However you do it, you need log **N** steps to compute the **∑**.

© 2022 Blum & Blum

Q7. Why is the resolution mechanism (in the Up-Tree competition) the specific one that is proposed?

A. For the probabilistic **CTM**, the decision made at each interior node of the **Up-Tree** – namely which one of the node's two children's chunks should win the match - is decided by which chunk has the larger **f**-value. As processor **p**'s chunk works its way up the tree, its **f**-value is affected by those processors that neighbor **p**. It is a surprising consequence of this mechanism that if **f** is additive then the probability that a chunk rises to **STM** is independent of its location, that is to say the location of the processor that generated it, on (the leaves of) the competition **Up-Tree**. As a consequence, the competition is permutation independent.

There are other completely different and even more important reasons for making **f** additive: for one, when **f** is not additive, in each and every one of the many nontrivial examples considered, something always goes terribly wrong. For example, if **f (chunk) = |mood|** then a strong positive mood and a strong negative mood appearing in two siblings, children of a node, completely cancel: neither "becomes" conscious (reaches **STM**) even when all other chunks are relatively unimportant. For example, consider the following **Up-Tree** with **f = |mood|** and **w_1 = 100, w_2 = -100, w_3 = 1, w_4 = 2**. Then the **Up-Tree competition** looks like this:

```
f = |mood|        Mood =              6  <—  Pr (A) = pr(B) = 0   Pr(C) = 1/3   Pr(D) = 2/3
                                    /   \
                  Mood =         0         6  <—  Pr(C) = 1/3   Pr(D) = 2/3
                               /   \      /  \
         Weight = Mood =     100  -100   2    4
                              A     B    C    D
```

For another example, if **f(chunk) = |weight| (**see figure in answer **A1** to **Q9)**, then two chunks having the same maximum **|weight|** can have vastly different probabilities of reaching **STM**. This does not happen if **f** is additive.

Q8. Why do you focus on the probabilistic rather than the deterministic CTM as being the correct model?

A. There are *many* reasons for this. For one, as noted above, with any additive competition function such as **f(chunk) = intensity**, the competition is permutation independent if **CTM** is probabilistic. This is not the case if **CTM** is deterministic, not even if **f** is additive:

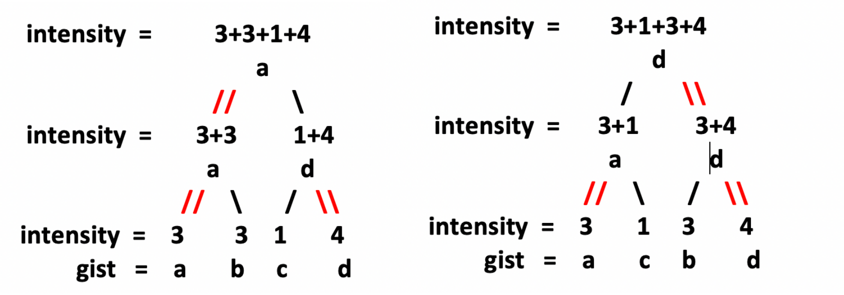

*Deterministic Competition Trees with Competition Function **f: chunk → intensity**.*

For another, in the probabilistic **CTM**, gists submitted to the competition, even those with small intensity, get into **STM** with probability proportional to their estimated importance (**f**-value). Again, this is not the case for a deterministic **CTM**.

© 2022 Blum & Blum

Q9. Why do the authors choose to have intensity and mood in the chunk? It seems equally valid to discard them.

A1. Without **intensity** and **mood** in the chunk, every "reasonable" competition function such as **f(chunk) = |weight|** is non-additive, which leads to the possibility of a weird lopsided kind of consciousness. For example, suppose the competition tree has **N/2** chunks each of a *heavy* **weight W** in the left-hand subtree (**LHST**), exactly **1** chunk of the same weight **W** in the right-hand subtree (**RHST**), and that all other chunks have *negligible* **weight**, as in the next figure. In that case, though **CTM** considers all heavily weighted chunks equally important, the (heavily weighted) chunks in the **LHST** each have negligible probability **1/N** to get into **STM**, while the single heavily weighted chunk in the **RHST** has probability almost **1/2** to get into **STM**:

```
weight =              100       <------ pr{A} = pr{B} = pr{C} = pr{D} ≈ 1/8;  pr{E} ≈ 1/2
                    /       \
weight =         100         100
                /   \       /   \
weight =      100    100   100    1
             /  \   /  \   / \   / \
weight =   100 100 100 100 100 1  1   1
            A   B   C   D   E  F  G   H     <------ N=8
```

A2. At time **t+h**, the winning chunk contains the **weight** that was originally assigned to it at time **t** when it got put into the competition. The **intensity** and **mood** of that winning chunk, which were set to **|weight|** and **weight** respectively at time **t**, got continuously modified as the chunk moved up the competition tree until the chunk entered the **STM** at time **t+h**, at which time the **intensity** and **mood** are indicators of (**N** times) the average intensity and mood of the entire **CTM** at time **t**. We believe that humans are normally consciously aware of their global intensity and mood, a fact that makes it entirely reasonable to include **intensity** and **mood** in the chunk.

A3. Chess and tennis tournaments use seeding to give players of equal strength roughly equal chances of winning the tournament. The **Up-Tree competition** with *additive* competition function assures that even without seeding, all players have a probability of winning proportional to their ability/expertise.

Q10. Where does feedback come from?

A. Feedback comes from chunks that are received in **broadcasts** from **STM**, through **links**, and from the environment via **Input maps**, all of which have information that can be graded as "erroneous" or "correct" in comparison to (stored) predictions.

Q11. How do processors judge whether or not their information is valuable?

A. Judgements are based on feedback. Each processor has a **Sleeping Experts Algorithm (SEA)** that learns, based on feedback, what the weight-giving power of its processor should have been, so that weight assignments eventually settle down to something more or less correct. Roughly speaking, when a **|weight|** is too low, the **SEA** multiplies the weight-giving power by **2** (more generally by some constant **c > 1**). When too high, the **SEA** multiplies it by ½ (more generally by **1/c**).

© 2022 Blum & Blum

Q12. Why would chunks contain queries and answers?

A. For example, when you meet a person at a party and can't remember her name, a chunk produced by a processor can pose the query "What's her name?". When this chunk wins the competition for **STM**, its query is broadcast to all **LTM** processors. Sometime later, another processor answers, "I think her name begins with **T**," which rises to **STM** and gets broadcast. This can later, perhaps much later, trigger another processor to answer, "Her name is **Tina**."

Q13: Must the CTM have a Model-of-the-World processor?

A1: The **Model-of-the-World processor** is a fundamental component of consciousness. Our explanation for the "feeling of consciousness" is for a **CTM** that has a **Model-of-the-World**. We don't see how to do without it.

A2. Our argument that **CTM** *feels* conscious depends on it having a **Model-of-the-World processor** and is akin to the argument given by the Attention Schema of (Graziano, Guterstam, Bio, & Wilterson, 2020).

We argue that **CTM's** *feeling* of consciousness, in the sense that the term is normally understood, is a consequence of the fact that what the **CTM** *consciously knows* (in the formal sense) of the world and of itself in the world *is* the **Model-of-the-World processor's** view of the world, and its view of the "**CTM**" in its **models of the world**; and that this view, which includes that the "**CTM**" is conscious, is broadcast to all processors.

Q14. Isn't naming specific processors, such as the Model-of-the-World processor, in definite and final terms, an over-specification of the model?

A. The proposed **Model-of-the-World processor** is only an example of how such a processor might work. It is not meant to be the definitive final specification. It's kind of like Turing's universal machine. The idea is important, as is having a description of such a machine. The particular machine is less important.

Q15. Why would the whole cortex not constitute a Model-of-the-World processor (as commonly assumed in neuroscience) and not just the Model-of-the-World processor?

A. The whole cortex may well be viewed as a model of the world in both the human brain and the **CTM**. We don't suggest otherwise.

Q16. Why would the lack of input from the environment lead to incoherent thoughts in dreams?

A. No, no, we're not saying that dreams *must* be incoherent, only that they *can* be incoherent, and that this is especially the case in dreams because the processors involved in a dream are not getting feedback from the environment. For example, in a dream, one might believe that one can fly.

Q17. What question does the theory address that is not already accounted for by the standard GWT-related theories?

A1. Unlike standard **GWT**-related theories of consciousness, **CTM** is a substrate independent computational model of consciousness, not a model of the brain. Its purpose is to explain how a machine can *experience* feelings. As Arlindo Oliveira has pointed out: "The proposed model is not a model of human consciousness, but a computational model that can explain many features of conscious behavior and that address directly the hard problem of consciousness, as defined by Chalmers. [It explains] why systems that are subject to the laws of physics can have subjective experiences."

A2. No other **GWT**-related theory gives a substantive idea how processors might decide among themselves what information to send to the stage.

© 2022 Blum & Blum

Q18. How does the theory argue that CTM has free will?

A. We don't. We argue that **CTM** has the *feeling* of free will. Our argument is two-fold.

The first part of the argument has to do with resource limitations - a complexity theory argument. For example, when the **CTM** plays chess, it can be faced with a selection of possible moves but, not yet having evaluated the consequences of those moves, the **CTM** is free (and knows it is free) to choose whichever move it reckons best - within the time constraints.

The second part of the argument is that **CTM**'s **Model-of-the-World processor** tags the "**CTM**" in its **models-of-the-world** with a multi-modal Brainish gist asserting that **CTM** is in the process of choosing its next move - meaning that it (the **CTM**) is free to choose its next move. Of course, its decision is deterministic (assuming as we do Newton's deterministic physics). However, this labeling of "**CTM**" as having **free will** gives **CTM** its knowledge that its processors may now suggest the next move, and this knowledge is conveyed by a *feeling of* **free will**. This argument is similar to our argument that **CTM** *feels* it is conscious.

Q19. Do you have an implementation of the CTM?

A. We do not. That said, Jean-Louis Villecroze is working on an implementation (Villecroze, 2019), and Paul Liang is working on developing the multi-modal language Brainish (Liang, 2022) in his PhD research on multi-modal machine learning. Our own focus is on understanding the hard problem of consciousness and some essential related issues. In this paper, we are not attempting to provide novel biological predictions, nor AI implementations - as wonderful as those would be - but to provide a simple machine model for consciousness.

We note that it took Alan Turing almost a decade (mostly due to his war work) to go from his theoretical one- tape universal machine to his complete circuit specification for the implementation of a universal computer – a description so complete that it included vacuum tube choices, resistor and capacitor values, mercury delay line memories, and even the cost of the computer in pounds (Turing A. M., 1945). Unfortunately, due to politics, Turing's Automatic Computing Engine (ACE) never saw the light of day; only a more primitive computer, the Pilot ACE, was constructed. (Hodges, 1992).

Q20. Why is there little mention of neural correlates of consciousness, particularly with respect to the phenomenal aspects of consciousness?

A1. The **CTM** is a computational substrate-independent model of consciousness, not a model of human or animal consciousness. (That said, a number of explanations from **CTM** of phenomenal aspects of consciousness such as blindsight are corroborated at a high level by cognitive neuroscience literature.)

A2. Even a complete knowledge of the "circuitry" of the brain and a complete knowledge of the neural correlates of consciousness – as wonderful and desirable as it would be to have these - cannot explain how the *feeling* of consciousness arises. To understand that feeling, something else is needed. That something is what we are proposing to get a handle on with the **CTM**.

Q21. Could brain dynamics and competition between attractors be a more neurally plausible explanation for how the brain works?

A. Perhaps, but... we're not looking to model the brain or brain dynamics but to understand consciousness. For this purpose, we use the mathematics that we find most helpful.

Q22. Assuming the brain is a CTM, what are some conditions for a part of the brain to be considered the STM?

A. The **STM** has a very small memory, a relatively small direct input, and an output that goes almost everywhere. In "A Brain Structure Looking for a Function" (Koch, 2014), Christof Koch suggests that the claustrum might fit the bill.

© 2022 Blum & Blum

3 Altered States of Consciousness (added section)

Under psychedelics or meditation, humans can experience altered states of consciousness ranging from a heightened sense of awareness to dissolution of self (feelings of being "one with the world"). We agree with (Bayne & Carter, 2018) that these are *states*, not levels, of consciousness. We disagree, however, with their assessment that the global/global neuronal workspace theories are too simple to explain these altered states. Indeed, the beauty of those theories lies in the significant understanding that comes of their simplicity.

Here we show how the **CTM** might experience a simple form of *dissolution of self*. We start by describing a **Mindful Meditation processor (MMp)** that a **CTM** might have.

The conscious decision to meditate would be the concern of the **MMp** that creates and submits a sequence of chunks to the competition for **STM**. Through repeated practice, this processor gains strength and increases the intensity of its chunks. It can be surprisingly difficult for the **MMp** to keep other chunks from entering **STM**. The difficulty is not in the sense of lifting a heavy weight or proving a difficult theorem, but in the sense of demanding focused concentrated attention and practice. (Rathi, 2021) explains how a human, using the *Mantra* meditation technique, accomplishes this.

When the **MMp** is successful, its chunks get into **STM** and are broadcast. Those broadcasts generally contain feedback that other processors use, through their **Sleeping Experts Algorithm**, to *hush* their own self-evaluations. Thus during successful meditation, the **MMp**'s chunks get the lion's share of time in **STM**.

Additionally, during successful meditation, chunks that get communicated via **links** from all processors except the **MMp** get hushed – by the incoming broadcasts from **MMp** – and thus processors are unlikely to pay their usual attention to the chunks they receive through links.[3]

This "hushing" or diminishing of functional connectivity is observed in studies on effects of psychedelics and meditation. For example, brain imaging and electromagnetic studies on effects of certain psychedelics (psilocybin) suggest that the dissolution of self ("ego-dissolution") is due to "disintegration" of functional connectivity (Calvey & Howells, 2018). This decreased connectivity accounts in part for the sense of dissolution of spatial boundaries, which in turn leads to the feeling of being "one with the world".

---

[3] During successful meditation, the hushing of chunks to be communicated via links diminishes link communication. This in effect diminishes the **Model-of-the-World** processor's ability to communicate to others what is **self** and what is **not-self**.

Neuroimaging studies on various forms of meditation from distinct traditions share some common neural correlates, see (Millière, Carhart-Harris, Roseman, Trautwein, & Berkovich-Ohana, 2018). Importantly, the latter report that in several forms of meditation there is "attenuation for either activity or functional connectivity" in the medial prefrontal cortex and in the posterior cingular cortex, key nodes of the so-called default mode network (DMN). The DMN is active when a person is daydreaming or mind-wandering. It is also active when a person is thinking about others or themselves, remembering the past, or planning for the future, see (Buckner, Andrews-Hanna, & Schacter, 2008) and (Lieberman, 2013). Thus attenuation of functional connectivity in these areas may also account for dissolution of self.

© 2022 Blum & Blum

4 Relation of CTM to Other Theories of Consciousness

The **CTM** is an abstract computational model designed to consider consciousness from a **TCS** perspective. It is not intended to model the brain nor the neural correlates of consciousness. Nevertheless, the **CTM** is both inspired by, and has certain features in common with, neural, cognitive, and philosophical theories of consciousness.

The **CTM** is directly influenced by Bernard Baars' **GWT** (Baars B. J., 1997), which is supported by (Dehaene & Changeux, 2011), (Dehaene S. , 2014) and (Mashour, Roelfsema, Changeux, & Dehaene, 2020) in their investigation of neural correlates of consciousness known as the Global Neuronal Workspace Theory (**GNWT**). We are inspired by David Mumford's 1991 work on the computational architecture of the neocortex (Mumford, 1991), which we view as an early proposal for **GNWT**.

Like the **LIDA** model of cognition (Baars & Franklin, 2007) and (Baars & Franklin, 2009), **CTM** is architectural. Unlike **LIDA**, which is a more elaborate model of **GWT**, the **CTM** is intended to be a *minimal* model of **GWT** sufficient to explain a wide range of conscious phenomena and, in particular, the *feeling* of consciousness.

Predictive dynamics (the ensemble of prediction, feedback, and learning) is an additional key feature of the **CTM**. It is related to the notion of predictive processing (**PP**), see (Lee & Mumford, 2003) (Friston, 2003) (Friston, 2005) (Cleeremans, 2014) (Clark, 2015) (Seth, 2015) (Hohwy & Seth, 2020).

We see a kinship between the **CTM** and the self-aware robots developed by (Chella, Pipitone, Morin, & Racy, 2020). We also see a kinship between the **CTM** and the Global Latent Workspace (**GLW**) proposed by (VanRullen & Kanai, 2021) for deep learning.

Our explanation for **CTM**'s "feeling of consciousness" aligns closely with Michael Graziano's Attention Schema Theory (**AST**) (Graziano, Guterstam, Bio, & Wilterson, 2020). As in **AST**, **CTM** is consciously aware of both external and internal events. Basic **AST** is similar to **GWT**: its i-consciousness (i for information) aligns somewhat with **CTM**'s conscious awareness.[4]

However, we do not agree with Graziano et al. that **GWT** "leaves unexplained how people end up believing they have subjective experience" i.e., that it leaves an explanatory gap. Instead, we argue that in our model, the feeling of subjective experience arises when "winning chunks" from imaginings and dreams, for example, are received by the same (unconscious) processors that receive chunks directly from the environment via **Input maps**. Additionally, the **Model-of-the-World processor** incorporates the information gotten from the winning chunks (i.e., the **conscious content** of the **CTM**) into its **models of the world**, as appropriate, tagging the "**CTM"** in all **models of the world** as "conscious". This is similar to Graziano's argument for consciousness in the **AST**. Fuller discussion for the *feeling* of consciousness in the **CTM** is in (Blum & Blum, 2021).

Philosophically, we align with much of Daniel Dennett's functionalist perspective (Dennett D. C., 1991) except we don't agree with his view that we are the only species to have consciousness (Dennett D. C., 1978) (Dennett D. C., 2019). As for animal consciousness, we agree with (Mumford, 2019) that consciousness is a matter of degree. Here he cites (Merker, 2007) that consciousness does not need a cerebral cortex: it arises from midbrain structures. We would also cite other studies, e.g., (Slobodchikoff, 2012).

We do not see the *explanatory gap* (Levine, 1983) between functional and phenomenological consciousness as insurmountable. This viewpoint aligns closely with Baars (see (Kaufman, 2020) interview) and (Dennett D. C.,

---

[4] Full **AST** has three neural networks (**A** for receiving information, **B** for constructing an attention schema, and **C** for reporting to the outside world) to obtain a system which purportedly thinks it has subjective experience (m-consciousness, m for mysterious).

© 2022 Blum & Blum

2016). Indeed, we see the **CTM**'s ability to tag and test features in its **models of the world** as playing a role in the feeling of "what it is like" (Nagel, 1974).

Both **AST** and **CTM** appear to embody illusionist notions of consciousness proposed by (Dennett D. C., 2019) and Keith Frankish (Frankish, 2016). Saying that the feeling of consciousness is an illusion does not deny the existence of that feeling. As a familiar example, the fact that a movie is made up of (many) discrete still images does not affect the feeling of continuity one gets from viewing it. The feeling of continuity is an illusion.

By utilizing existing technology (or apps) to supplement its supply of **LTM** processors, **CTM** incorporates elements similar to those advocated by (Clark & Chalmers, 1998)'s "extended minds".

Integrated Information Theory (**IIT**), the theory of consciousness developed by Giulio Tononi, (Tononi, 2004) and supported by Koch (Tononi & Koch, 2015), proposes a measure of consciousness called **PHI**, inspired by Shannon's information theory that essentially measures the amount of feedback in a system. It is a mechanism's intrinsic ability to influence itself, rather than its input-output information processing, that determines its consciousness.

This is consistent with **CTM**'s *intrinsic* predictive dynamics (of prediction, feedback and learning). Tononi proposes five "axioms" (properties) necessary for any causal system to have consciousness.[5] Given a detailed specification of a **CTM**, one could in principle compute its **PHI** and compare it to the **PHI** of any other precisely defined causal system. It turns out that many causal physical systems have non-zero measures of **PHI**. **IIT** would validate animal consciousness.

With regard to the "adversarial collaboration" between advocates of **GNWT** and **IIT**, (Reardon, 2019) and (Melloni, Mudrik, Pitts, & Koch, 2021), the **CTM** shares features of both basic theories, as pointed out above. Our view is that both theories add to the discussion of consciousness. The adversarial aspects between the theories arise mainly from the advocates' differing views on brain regions primarily responsible for consciousness –prefrontal cortex for **GNWT**, posterior cortex for **IIT**. We note however, it is possible to have some **level** of consciousness without a cerebral cortex at all (Merker, 2007) and suspect that in such cases, as in the **CTM**, aspects of the basic **GWT** and **IIT** are still in play.

Our view on **free will** is close to Dehaene's (Dehaene S. , 2014). Our explanation of the *feeling* of free will in the **CTM** incorporates additionally and *especially,* resource limits imposed by computational complexity considerations.

---

[5] In (Koch, 2019), Christof Koch outlines the axioms: "[E]very conscious experience has five distinct and undeniable properties: each one exists for itself, is structured, informative, integrated and definite".

© 2022 Blum & Blum

5 About the Authors of the expanded monograph (in progress)

Manuel has been motivated to understand the mind/body problem since he was in second grade when his teacher told his mom she should not expect him to get past high school. As an undergrad at MIT, he spent a year studying Freud and then apprenticed himself to the great anti-Freud[6] neurophysiologist, Dr. Warren S. McCulloch, who became his intellectual mentor. When he told Warren (McCulloch) and Walter (Pitts) that he wanted to study consciousness, he was told in no uncertain terms that he was verboten to do so and why (there was no fMRI at the time). As a graduate student, he asked and got Marvin Minsky to be his thesis advisor. Manuel is one of the founders of complexity theory, a Turing Award winner, and has mentored many in the field who have chartered new directions ranging from computational learning, cryptography, zero knowledge, interactive proofs, proof checkers, and human computation. He is a Fellow of $AAAS_1$, $AAAS_2$, NAS, NAE. **Manuel Blum** mblum@cs.cmu.edu

Lenore has been passionate about mathematics since she was 10. She attributes that to having dropped out of school when she was 9 to wander the world, then hit the ground running when she returned and became fascinated with the Euclidean Algorithm. Her interests turned to non-standard models of mathematics, and of computation. As a graduate student at MIT, she showed how to use saturated model theory to get new results in differential algebra. Later, with Mike Shub and Steve Smale, she developed a foundational theory for computing and complexity over continuous domains such as the real or complex numbers. The theory generalizes the Turing-based theory (for discrete domains) and has been foundaional for computational mathematics.

Lenore is internationally known for her work in increasing the participation of girls and women in STEM and is proud that CMU has gender parity in its undergraduate CS program. Over the years, she has been active in the mathematics community: as President of the Association for Women in Mathematics, Vice-President of the American Mathematical Society, Chair of the Mathematics Section of the American Association for the Advancement of Science, Deputy Director of the Mathematical Sciences Research Institute, and as Inaugural and current President of the Association for Mathematical Consciousness Science (AMCS). She is a Fellow of AAAS, AMS, AWM. **Lenore Blum** lblum@cs.cmu.edu

Avrim had an earlier start than the elder Blums. He spent his first two years at MIT, in his mom's office in the Math Department, and in his dad's office in McCulloch's lab. In sixth grade, he solved an extra credit math problem by programming his home-made computer to get a feel for the problem, then (once he saw what was going on) stated and proved the desired result. Because he used a computer, he got no credit. Odd, because he was pointing to a novel way (at the time) to solve a math problem. Avrim's expertise is Machine Learning Theory. He has been an advisor to many of the young leaders in the field.

Avrim is an active member of the computer science community. He has served as Program Chair for the IEEE Symposium on Foundations of Computer Science (FOCS), the Innovations in Theoretical Computer Science Conference (ITCS), and the Conference on Learning Theory (COLT). He has served as Chair of the ACM SIGACT Committee for the Advancement of Theoretical Computer Science and on the SIGACT Executive Committee. He is recipient of the AI Journal Classic Paper Award, the ICML/COLT 10-Year Best Paper Award, a Sloan Fellowship, the NSF National Young Investigator Award, and the Herbert Simon Teaching Award. He is a Fellow of the ACM. **Avrim Blum** avrim.blum@gmail.com

----

All three Blums received their PhDs at MIT and spent a cumulative 65 wonderful years on the faculty of the Computer Science Department at CMU. Currently the elder two are emeriti and the younger is Professor and Chief Academic Officer at TTIC (Toyota Technological Institute at Chicago), a PhD-granting computer science research institute focusing on areas of machine learning, algorithms, AI (robotics, natural language, speech, and vision), data science and computational biology, and located on the University of Chicago campus. Manuel Blum is Emeritus Professor of Computer Science at UC Berkeley and CMU. Lenore Blum is Emerita Distinguished Career Professor of Computer Science at CMU and is currently a Distinguished Professor-in-Residence at UC Berkeley.

---

[6] Where Freud had written *The Future of an Illusion* (Freud S. , 1927) , McCulloch followed with "The Past of a Delusion" (McCulloch, 1953).

© 2022 Blum & Blum

6 Figures

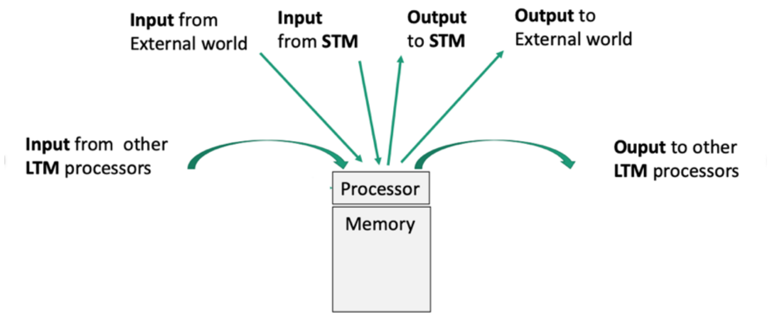

*Connections in **CTM** to and from an **LTM processor.***

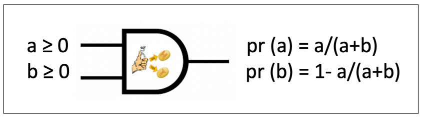

A coin-flip neuron on input (a, b) with a + b > 0.

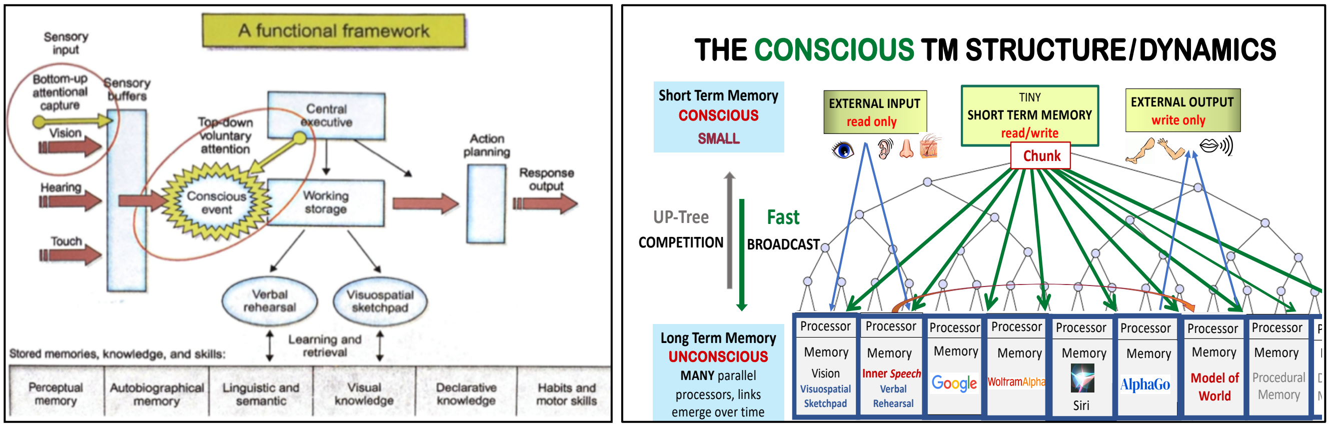

*Baars' **GWT** model (l); **CTM** (r).*

© 2022 Blum & Blum

Selective attention test:

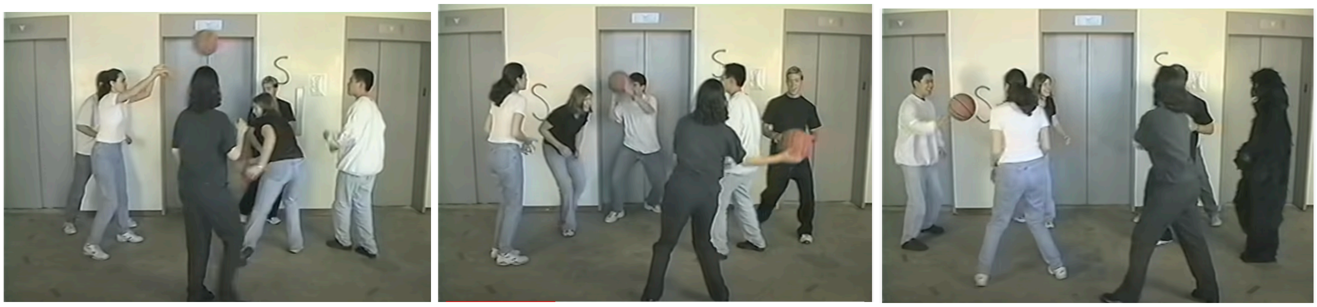

Screen shots from video, "The original selective attention task" (Chabris & Simons, 1999).

The *Whodunnit* video:

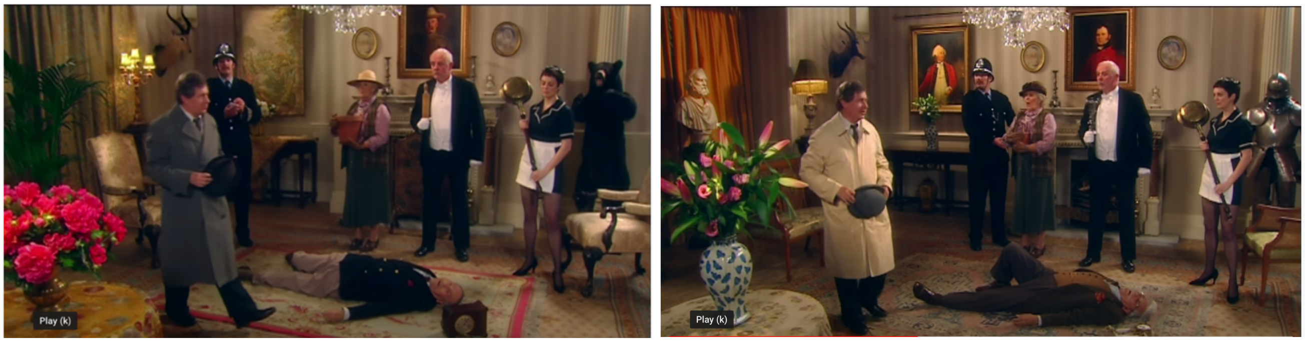

Beginning and ending screen shots from video (London, 2008).

© 2022 Blum & Blum

7 References

Ajina, S., & Bridge, H. (2018). Blindsight relies on a functional connection between hMT+ and the lateral geniculate nucleus, not the pulvinar. PLoS Biol 16(7): e2005769. https://doi.org/10.1371/journal.pbio.2005769. *PLoS Biol, 16*(7).

Al Roumi, F., Marti, S., Wang, L., Amalric, M., & Dehaene, S. (2020, November 20). *Mental compression of spatial sequences in human working memory using numerical and geometrical primitives* . Retrieved from bioRxiv: https://doi.org/10.1101/2020.01.16.908665

Anderson, J. R. (1996). ACT: A simple theory of complex cognition. *American Psychologist, 51(4)*, 355-365.

Baars, B. J. (1988). *A Cognitive Theory of Consciousness.* Cambridge: Cambridge University Press.

Baars, B. J. (1997). *In the Theater of Consciousness.* New York: Oxford University Press.

Baars, B. J. (1997). In the Theater of Consciousness: A rigorous scientific theory of consciousness. *Journal of Consciousness Studies 4, No. 4*, 292-309.

Baars, B. J. (2019). *ON CONSCIOUSNESS: Science & Subjectivity - Updated Works on Global Workspace Theory.* New York: Nautilus Press.

Baars, B. J., & Franklin, S. (2007, November). An architectural model of conscious and unconscious brain functions: Global Workspace Theory and IDA. *Neural Networks Special Issue, 20*(9), 955-961. Retrieved from https://doi.org/10.1016/j.neunet.2007.09.013

Baars, B. J., & Franklin, S. (2009). Consciousness is computational: The LIDA model of Global Workspace Theory. *International Journal of Machine Consciousness, 1(1)*, 23-32.

Baddeley, A. D., & Hitch, G. J. (1974). Working memory. In G. A. Bower (Ed.), *The Psychology of Learning and Motivation* (pp. 47-89). New York: Academic Press.

Bayne, T., & Carter, O. (2018, September 19). Dimensions of consciousness and the psychedelic state. *Neuroscience of consciousness,*.

Blum, A. (1995, July). Empirical support for winnow and weighted-majority algorithms: Results on a calendar scheduling domain. (A. Prieditis, & S. J. Russell, Eds.) *Proceedings of the Twelfth International Conference on Machine Learning*, 64-72.

Blum, A. (1997). Empirical support for winnow and weighted-majority algorithms: Results on a calendar scheduling domain. *Machine Learning, 26*(1), 5-23.

Blum, A., & Mansour, Y. (2007). From external to internal regret. *Journal of Machine Learning Research*, 1307-1324.

Blum, A., Hopcroft, J., & Kannan, R. (2015). *Foundations of Data Science.* Ithaca. Retrieved from https://www.cs.cornell.edu/jeh/book.pdf

Blum, L., & Blum, M. (2022). *A Theory of Consciousness from a Theoretical Computer Science Perspective: Insights from the Conscious Turing Machine*. Retrieved May 2022, from arXiv: arxiv.org/pdf/2107.13704.pdf

Blum, M., & Blum, L. (2021, March). A Theoretical Computer Science Perspective on Consciousness. *JAIC, 8*(1), 1-42. https://www.worldscientific.com/doi/epdf/10.1142/S2705078521500028.

Boswell, J. (1791). *The Life of Samuel Johnson.* Poultry, London, England: Printed by Henry Baldwin for Publisher: Charles Dilly.

Buckner, R. L., Andrews-Hanna, J. R., & Schacter, D. L. (2008, April 3). The Brain's Default Network: Anatomy, Function, and Relevance to Disease. *1124*(1), 1-38. https://doi.org/10.1196/annals.1440.011 .

Calvey, T., & Howells, F. M. (2018). An introduction to psychedelic neuroscience. . In T. (. Calvey, *Psychedelic Neuroscience, Progress in Brain Research* (Vol. 242, pp. 1-23). Elsevier.

Carhart-Harris, R., Muthukumaraswamy, S., Roseman, L., Kaelen, M., Droog, W., Murphy, K., . . . Leech, R. (2016, April). Neural correlates of the LSD experience revealed by multimodal neuroimaging Proc. Natl. A. *PNAS, 113*(17), 4853–4858.

Chabris, C., & Simons, D. (1999). *The Invisible Gorilla*. Retrieved May 7, 2022, from http://www.theinvisiblegorilla.com/videos.html

Chalmers, D. J. (1995). Facing Up to the Problem of Consciousness. *Journal of Consciousness Studies, 2*(3), 200-219.

Chalmers, D. J. (1996). *The Conscious Mind: In Search of a Fundamental Theory.* New York: Oxford University Press.

Chella, A., Pipitone, A., Morin, A., & Racy, F. (2020, February). Developing Self-Awareness in Robots via Inner Speech . *Frontiers in Robotics and AI, 7*. Retrieved from https://www.frontiersin.org/article/10.3389/frobt.2020.00016

Church, A. (1936, April). An Unsolvable Problem of Elementary Number Theory. *American Journal of Mathematics , 58*(2), 345-363.

© 2022 Blum & Blum

Clark, A. (2015). Embodied prediction. In T. Metzinger, & J. Windt, *Open Mind.* Frankfurt am Main: MIND Group.
Clark, A., & Chalmers, D. (1998, January). The Extended Mind. *Analysis, 58*(a), 7-19.
Cleeremans, A. (2014). Prediction as a computational correlate of consciousness. *International Journal of Anticipatory Computing Systems, 29*, 3-13.
Collins Dictionary. (2020). Retrieved May 2020, from https://www.collinsdictionary.com/us/dictionary/english/free-will
Cook, S. A. (1971). The complexity of theorem-proving procedures. *Proceedings of the third annual ACM symposium on Theory of computing. 8https://doi.org/10.1145/800157.805047*, pp. 151-158. New York: Association for Computing Machinery.
Corlett, P. R., Canavan, S. V., Nahum, L., Appah, F., & Morgan, P. T. (2014). Dreams, reality and memory: confabulations in lucid dreamers implicate reality-monitoring dysfunction in dream consciousness. . *Cognitive neuropsychiatry, 19*(6), 540–553.
Crick, F., & Koch, C. (1990). Towards a neurobiological theory of consciousness. *Seminars in Neuroscience 2*, 263–275.
Dawson, J. W. (1997). *Logical Dilemmas: The Life and Work of Kurt Gödel.* Wellesley, MA, USA: A. K. Peters.
Dehaene, S. (2014). *Consciousness and the Brain: Deciphering How the Brain Codes Our Thoughts.* New York: Viking Press.
Dehaene, S., & Changeux, J. P. (2005). Ongoing Spontaneous Activity Controls Access to Consciousness: A Neuronal Model for Inattentional Blindness. . *PLoS Biol, 3*(5).
Dehaene, S., & Changeux, J. P. (2011). Experimental and theoretical approaches to conscious processing. *Neuron; 70(2)*, 200-227.
Dehaene, S., & Changeux, J.-P. (2005). Ongoing Spontaneous Activity Controls Access to Consciousness: A Neuronal Model for Inattentional Blindness. *PLoS Biol, 3*(5).
Dehaene, S., Changeux, J. P., & Naccache, L. (2011). The Global Neuronal Workspace Model of Conscious Access: From Neuronal Architectures to Clinical Applications. In S. Dehaene, & Y. (. Christen, *Characterizing Consciousness: From Cognition to the Clinic?. Research and Perspectives in Neurosciences.* Berlin, Heidelberg: Springer-Verlag.
Dennett, D. C. (1978). Why You Can't Make a Computer That Feels Pain. *Synthese Vol. 38, No. 3, Automaton-Theoretical Foundations of Psychology and Biology, Part I*, 415-456.
Dennett, D. C. (1991). *Consciousness Explained.* Boston; Toronto; London: Little, Brown and Co.
Dennett, D. C. (2016). Illusionism as the Obvious Default Theory of Consciousness. *Journal of Consciousness Studies, 23*(11-12), 65-72.
Dennett, D. C. (2018, July 30). Facing up to the hard question of consciousness. *Philosophical transactions of the Royal Society of London. Series B, Biological sciences, 373*(1755). Retrieved from http://dx.doi.org/10.1098/rstb.2017.0342
Dennett, D. C. (2019, December). Consciousness, Qualia and the "Hard Problem". (L. Godbout, Interviewer) Retrieved from https://youtu.be/eSaEjLZIDqc, starting time for quote 5:40
Edmonds, J. (1965). Paths, trees, and flowers. *Can. J. Math., 17*, 449-467.
Epstein, R. A. (2008). Parahippocampal and retrosplenial contributions to human spatial navigation. *Trends in cognitive sciences , 12*(10), 388-396 doi:10.1016/j.tics.2008.07.004.
Frankish, K. (2016). Illusionism as a Theory of Consciousness,. *Journal of Consciousness Studies, 23*((11-12)), 11-39.
Freud, S. (1900). *The Interpretation of Dreams* (Vols. IV–V, Standard Edition). London: Hogarth.
Freud, S. (1927). *The Future of an Illusion.*
Freund, Y., Schapire, R. E., Singer, Y., & Warmuth, M. K. (1999). Using and combining predictors that specialize. *Proc. 29th Annual ACM Symposium on the Theory of Computing.*
Fried, I., Mukamel, R., & Kreiman, G. j. (2011, February 10). Internally Generated Preactivation of Single Neurons in Human Medial Frontal Cortex Predicts Volition. *Neuron, 69*(3), 548-562.
Friston, K. (2003). Learning and inference in the brain. *Neural Networks, 16*(9), 1325-1352.
Friston, K. (2005, April 29). A theory of cortical responses. *Phil. Trans. R. Soc. B doi:10.1098/rstb.2005.1622, 360*, 815-836.
Gödel, K. (1931). Uber formal unentscheidbare Satze der Principia Mathematica und verwandter Systeme I. *Monatshefte fur Mathematik und Physik, 38*, 173-198.
Gershman, S., Horvitz, E., & Tenenbaum, J. (2015, July 16). Computational Rationality: A Converging Paradigm for Intelligence in Brains, Minds, and Machines. *Science , 349*(6245), 273-278.
Gholipour, B. (2019, March 21). *Philosophers and neuroscientists join forces to see whether science can solve the mystery of free will.* Retrieved from ScienceMag.org: https://www.sciencemag.org/news/2019/03/philosophers-and-neuroscientists-join-forces-see-whether-science-can-solve-mystery-free

© 2022 Blum & Blum

Goldreich, O. (2010). *P, NP, and NP-Completeness.* Cambridge, England: Cambridge University Press; https://www.wisdom.weizmann.ac.il/~oded/bc-drafts.html. Retrieved from P versus NP problem: https://en.wikipedia.org/wiki/P_versus_NP_problem

Goodfellow, I., Bengio, Y., & Courville, A. (2016). *Deep Learning.* Cambridge, MA: MIT Press.

Graziano, M. S., Guterstam, A., Bio, B., & Wilterson, A. (2020, May-June). Toward a standard model of consciousness: Reconciling the attention schema, global workspace, higher-order thought, and illusionist theories. *Cognitive Neuropsychology, 37*(3-4), 155-172. Retrieved from doi:10.1080/02643294.2019.1670630

Graziano, M. S., Guterstam, A., Bio, B., & Wilterson, A. (2020, May-June). Toward a standard model of consciousness: Reconciling the attention schema, global workspace, higher-order thought, and illusionist theories. *Cognitive Neuropsychology, 37*(3-4), 155-172. Retrieved from doi:10.1080/02643294.2019.1670630

Haggard, P. (2011, February 11). Decision Time for Free Will6. *Neuron, 69*(3), 404-406.

Hebb, D. O. (1949). *The Organization of Behavior.* New York: Wiley & Sons.

Herlin, B., Leu-Semenescu, S., Chaumereuil, C., & Arnulf, I. (2015, December). Evidence that non-dreamers do dream: a REM sleep behaviour disorder model. *J Sleep Res., 24*(6), 602-609.

Hodges, A. (1992). *Alan Turing: The Enigma.* Vintage.

Hohwy, J., & Seth, A. (2020). *Predictive processing as a systematic basis for identifying the neural correlates of consciousness (preprint).* Retrieved May 7, 2022, from PsyArXiv: https://psyarxiv.com/nd82g

Horikawa, T., Tamaki, M., Miyawaki, Y., & Kamitani, Y. (2013, May 3). Neural Decoding of Visual Imagery During Sleep. *Science, 340*(6132), 639-642.

James, W. (1890). *The Principles of Psychology (2 vols.).* New York: Henry Holt (Reprinted Bristol: Thoemmes Press, 1999).

Jensen, M., Yao, R., Street, W., & Simons, D. (2011). Change blindness and inattentional blindness. *WIREs Cogn Sci, 2*, 529-546.

Karp, R. M. (1972). Reducibility Among Combinatorial Problems. In R. E. Miller, J. W. Thatcher, (eds.), R. E. Miller, & J. W. (eds.) (Eds.), *Complexity of Computer Computations* (pp. 85- 103). New York: Plenum.

Kaufman, S. B. (2020, May 26). *On Consciousness: Science and Subjectivity: A Q&A with Bernard Baars*. Retrieved from https://blogs.scientificamerican.com/beautiful-minds/on-consciousness-science-and-subjectivity-a-q-a-with-bernard-baars/

Koch, C. (2014, November). A Brain Structure Looking for a Function" in SA Mind 25, 6, 24-27 (November 2014)],. *SA Mind, 25*(6), pp. 24-27.

Koch, C. (2019). *The Feeling of Life Itself: Why Consciousness Is Widespread but Can't Be Computed.* Cambridge: MIT Press.

Kringelbach, M., & Berridge, K. C. (2017). The Affective Core of Emotion: Linking Pleasure, Subjective Well-Being, and Optimal Metastability in the Brain. *Emot Rev. doi:10.1177/1754073916684558, 9*(3), 191-199.

Lavazza, A. (2019, February 26). Why Cognitive Sciences Do Not Prove That Free Will Is an Epiphenomenon. *Front. Psychol., https://doi.org/10.3389/fpsyg.2019.00326*.

Lee, J. L., Nader, K., & Schiller, D. (2017). An Update on Memory Reconsolidation Updating . *Trends in Cognitive Sciences, 21*(7), 531-545.

Lee, T. S., & Mumford, D. (2003). Hierarchical Bayesian inference in the visual cortex. *Journal of the Optical Society of America, Optics, image science and vision, 20*(7), 1434-1448.

Lettvin, J., Maturana, H., McCulloch, W., & Pitts, W. (1959, November). What the Frog's Eye Tells the Frog's Brain. *Proceedings of the IRE, 47*(11).

Levin, L. A. (1973). Universal Sequential Search Problems. *Probl. Peredachi Inf., 9*(3), 115-116.

Levine, J. (1983). Materialism and Qualia: The Explanatory Gap. *Pacific Philosophical Quarterly, 64*, 354–361.

Liang, P. P. (2022, May 3). *Brainish: Formalizing A Multimodal Language for Intelligence and Consciousness.* Retrieved May 8, 2022, from arXiv: https://arxiv.org/abs/2205.00001

Libet, B. (1985). Unconscious cerebral initiative and the role of conscious will in voluntary action. *Behavioral and Brain Sciences, 8*(4), 529-539.

Lieberman, M. D. (2013). *Social: Why our brains are wired to connect.* New York, NY: Crown.

London, M. o. (2008, November 3). *Test Your Awareness : Whodunnit?* Retrieved May 7, 2022, from YouTube: https://www.youtube.com/watch?v=ubNF9QNEQLA&list=PLr4EeJcghrfSnnBO8YFu0qnz4IrpQEUaZ&index=1

Lucretius, & Ferguson Smith (translator), M. (1969). *On the Nature of Things.* Hackett Publishers.

Luo, H., & Schapire, R. E. (2015). Achieving all with no parameters: Adanormal-hedge. *Conference on Learning Theory*, (pp. 1286-1304).

© 2022 Blum & Blum

Maoz, U., Yaffe, G., Koch, C., & Mudrik, L. (2019, October 23). Neural precursors of decisions that matter-an ERP study of deliberate and arbitrary choice. *eLife, 8*(doi:10.7554/eLife.39787).

Mashour, G. A., Roelfsema, P., Changeux, J.-P., & Dehaene, S. C. (2020). Conscious Processing and the Global Neuronal Workspace Hypothesis. *Neuron, 195*(5), 776-798.

McCulloch, W. S. (1953, October). *The Past of a Delusion.* The Chicago Literacy Club.

Melloni, L., Mudrik, L., Pitts, M., & Koch, C. (2021, May 28). Making the hard problem of consciousness easier. *Science, 372*(6545), 911-912.

Merker, B. (2007). Consciousness without a cerebral cortex: a challenge for neuroscience and medicine. *Behav. Brain Sci. 30*, 63-81.

Merriam Webster . (2020).

Metzinger, T. (2004). The subjectivity of subjective experience: A representationalist analysis of the first-person perspective. *Networks, 3-4*, 33-64.

Miller, G. A. (1956). The Magical Number Seven, Plus or Minus Two: Some Limits on our Capacity for Processing Information. *Psychological Review, 63*, 81-97.

Millière, R., Carhart-Harris, R. L., Roseman, L., Trautwein, F.-M., & Berkovich-Ohana, A. T.-C. (2018). Psychedelics, Meditation, and Self-Consciousness. *Frontiers in Psychology , 9*.

Mumford, D. (1991). On the computational architecture of the neocortex. *Biological Cybernetics, 65*, 135-145.

Mumford, D. (2019). *Thoughts on Consciousness.* Retrieved May 7, 2022, from hhttps://www.dam.brown.edu/people/mumford/beyond/papers/2019--Thoughts%20on%20ConsciousnessB.pdf

Nagel, T. (1974). What Is It Like To Be a Bat? *Philosophical Review, 83*, 435–450.

Newell, A. (1990). *Unified Theories of Cognition.* Cambridge: Harvard University Press.

Paulson, S. (2017, April 6). The Spiritual, Reductionist Consciousness of Christof Koch. *Nautilus*. Retrieved from http://nautil.us/issue/47/consciousness/the-spiritual-reductionist-consciousness-of-christof-koch

Rathi, N. (2021). *Explaining the Process of Meditation via the Blum Model of Conscious Turing Machine.* Indian Institute fo Science, Computer Sciemce and Automation, Bangalore.

Reardon, S. (2019, October 16). *Outlandish' competition seeks the brain's source of consciousness.* Retrieved from sciencemag.org: doi:10.1126/science.aaz8800

Reddy, D. R. (1976, April). Speech Rcogniton by Machine: A Review. *Proceedings of the IEEE*, 501-531. Retrieved from http://www.rr.cs.cmu.edu/sr.pdf

Rensink, R. A., O'Regan, J. K., & Clark, J. J. (1997). To See or not to See: The Need for Attention to Perceive Changes in Scenes. *Psychological Science, 8*(5), 368-373.

Rosen, C., McCarthy-Jones, S., Chase, K. A., Humpston, C., Melbourne, J. K., Kling, L., & Sharma, R. P. (2018, June). The tangled roots of inner speech, voices and delusions. *Psychiatry Res.*, 281-289.

Ruch, S., Züst, M. A., & Henke, K. (2016, August 20). Subliminal messages exert long-term effects on decision-making. *Neuroscience of Consciousness*(https://doi.org/10.1093/nc/niw013).

Scarpelli, S., Bartolacci, C., D'Atri, A., Gorgoni, M., & De Gennaro, L. (2019, March). The Functional Role of Dreaming in Emotional Processes. *Frontiers in psychology, 10*.

Schurger, A., Sitt, J. D., & Dehaene, S. (2012, October 16). An accumulator model for spontaneous neural activity prior to self-initiated movement. *PNAS, 109*(42), E2904-E2913.

Seth, A. K. (2015). The Cybernetic Bayesian Brain - From Interoceptive Inference to Sensorimotor Contingencies. In T. T. Metzinger, & J. M. Windt, *Open MIND.* Frankfurt am Main: MIND Group. doi: 10.15502/9783958570108 23 | 24.

Simon, H. A. (1969). *The Sciences of the Artificial.* Cambridge, MA, USA: MIT Press.

Simons, D., & Chabris, C. (1999). Gorillas in our midst: sustained inattentional blindness for dynamic events. *Perception, 28*(9), 1059-74.

Sipser, M. (2013). *Introduction to the Theory of Computation, 3rd edition.* Boston: Cengage Learning.

Slobodchikoff, C. N. (2012). *Chasing Doctor Dolittle: Learning the Language of Animals.* New York: St. Martin's Press.

Striemer, C. L., Chapman, C. S., & Goodale, M. A. (2009, September 15). 'Real-time' Obstacle Avoidance in the Absence of Primary Visual Cortex. *Proc Natl Acad Sci U S A*.

Tononi, G. (2004). An information integration theory of consciousness. *BMC Neuroscience 5*, 42-72.

Tononi, G., & Koch, C. (2015). Consciousness: here, there and everywhere? *Philosophical Transactions of the Royal Society of London B: Biological Sciences, 370 (1668)*.

Turing, A. M. (1937). On Computable Numbers, with an Application to the Entscheidungsproblem. *Proceedings of the London Mathematical Society, 2*, 230-265.

© 2022 Blum & Blum

Turing, A. M. (1945). *Proposal for development in the Mathematics Division of an Automatic Computing Engine (ACE). .* Report E.882, Executive Committee, NPL, Mathematics.
Valiant, L. (2013). *Probably Approximately Correct.* New York: Basic Books Perseus Group.
VanRullen, R., & Kanai, R. (2021, May 14). Deep learning and the Global Workspace Theory. *Trends in Neurosciences*.
Villecroze, J.-L. (2019, June 16). *An Introduction to Conscious Turing Machines with fizz*. Retrieved from https://f1zz.org/downloads/ctm.pdf.
von Neumann, J. (1951). Various Techniques Used in Connection with Random Digits. In A. S. Householder, G. E. Forsythe, & H. H. Germond (Ed.), *Monte Carlo Method. 12*, pp. 48-50. Washington, DC: United States Government Printing Office.
Vyazovskiy, V., Delogu, & A. (2014, June). NREM and REM Sleep: Complementary Roles in Recovery after Wakefulness. *Neuroscientist, 20*(3), 203-219.
Wikipedia. (n.d.). *History of artificial neural networks*. Retrieved March 15, 2022, from https://en.wikipedia.org/wiki/History_of_artificial_neural_networks
Yao, A. C.-C. (1982). Theory and applications of trapdoor functions. *Proceedings of the 23rd IEEE Symposium on Foundations of Computer Science. FOCS '82*, pp. 80-91. IEEE Computer Society.
Yao, A. C.-C. (2003, January). Classical physics and the Church--Turing Thesis . *Journal of the ACM, 50*(1), 100-105.
Zadra, A., & Stickgold, R. (2021). *When Brains Dream Exploring the Science and Mystery of Sleep.* New York: W. W. Norton.

© 2022 Blum & Blum
